# Novel Isolation Method Reveals Sex-Specific Composition and Neurotoxicity of Small Extracellular Vesicles in a Mouse Model of Alzheimer’s Disease

**DOI:** 10.3390/cells12121623

**Published:** 2023-06-14

**Authors:** Ahmed Elsherbini, Zhihui Zhu, Zainuddin Quadri, Simone M. Crivelli, Xiaojia Ren, Hemendra J. Vekaria, Priyanka Tripathi, Liping Zhang, Wenbo Zhi, Erhard Bieberich

**Affiliations:** 1 Department of Physiology, University of Kentucky College of Medicine, Lexington, KY 40536, USA; aelsherbini@uky.edu (A.E.); zzh295@uky.edu (Z.Z.); zainuddin.quadri@uky.edu (Z.Q.); s.crivelli@uky.edu (S.M.C.); xiaojia.ren@uky.edu (X.R.); priyanka.tripathi@uky.edu (P.T.); lzhanh@uky.edu (L.Z.); 2 Spinal Cord and Brain Injury Research Center (SCoBIRC), University of Kentucky, Lexington, KY 40536, USA; hemendravekaria@uky.edu; 3 Veterans Affairs Medical Center, Lexington, KY 40502, USA; 4 Department of Center for Biotechnology and Genomic Medicine, Augusta University, Augusta, GA 30912, USA; wzhi@augusta.edu

**Keywords:** extracellular vesicles, Alzheimer’s, acid sphingomyelinase, ceramide, neurotoxicity

## Abstract

We developed a new method to isolate small extracellular vesicles (sEVs) from male and female wild-type and 5xFAD mouse brains to investigate the sex-specific functions of sEVs in Alzheimer’s disease (AD). A mass spectrometric analysis revealed that sEVs contained proteins critical for EV formation and Aβ. ExoView analysis showed that female mice contained more GFAP and Aβ-labeled sEVs, suggesting that a larger proportion of sEVs from the female brain is derived from astrocytes and/or more likely to bind to Aβ. Moreover, sEVs from female brains had more acid sphingomyelinase (ASM) and ceramide, an enzyme and its sphingolipid product important for EV formation and Aβ binding to EVs, respectively. We confirmed the function of ASM in EV formation and Aβ binding using co-labeling and proximity ligation assays, showing that ASM inhibitors prevented complex formation between Aβ and ceramide in primary cultured astrocytes. Finally, our study demonstrated that sEVs from female 5xFAD mice were more neurotoxic than those from males, as determined by impaired mitochondrial function (Seahorse assays) and LDH cytotoxicity assays. Our study suggests that sex-specific sEVs are functionally distinct markers for AD and that ASM is a potential target for AD therapy.

## 1. Introduction

Extracellular vesicles (EVs) are released by all cell types, including central nervous system cells, as a means of intercellular communication [1,2]. EVs’ ability to carry and deliver biological molecules such as lipids, proteins, RNA, and DNA fragments inspired researchers to isolate them from bodily fluids and tissues to study their physiological and pathological roles [3]. In the nervous system, a wide range of physiological and pathological functions have been attributed to EVs [4]. From a physiological perspective, EVs have been reported to contribute to neural development and neuronal activity [5]. On the other hand, several studies implicate EVs in neurodegenerative diseases such as Alzheimer’s disease (AD), Parkinson’s disease (PD), and amyotrophic lateral sclerosis [5,6]. It has been hypothesized that EVs may traffic misfolded proteins, such as amyloid-beta peptide (Aβ) and tau in AD and α-synuclein in PD between cells, thereby facilitating the spread of pathology and diseases throughout the brain [7,8,9,10,11].

However, understanding the full magnitude of the functions of EVs in the nervous system has been hindered by technical difficulties in isolating pure EVs from brain tissues. A major concern when isolating EVs is maintaining cell integrity. While there have been several reports of EV isolation methods using tissue homogenization and filtration, the chances of co-isolation of EV mimics such as subcellular organelles, myelin debris, and intracellular vesicles remain a pressing issue. Therefore, we report a method that comprises automated gentle enzymatic dissociation of brain tissues while ensuring the integrity of brain cells. EVs are then recovered from the extracellular matrix. EVs were rigorously characterized for their size, zeta potential, morphology, protein, and lipid (ceramide) composition. Furthermore, immunocapturing and analysis of EVs at the single-vesicle level were achieved using ExoView tetraspanin chips.

Previously, we demonstrated that the sphingolipid ceramide enriched in astrocyte-derived EVs plays a crucial role in increasing the neurotoxicity of Aβ, especially by intensifying mitochondrial damage in neurons [12]. However, our studies indicated that there is a sexual dimorphism in the impact of ceramide-enriched EVs [13]. In male 5xFAD mice, pharmacological inhibition or genetic deficiency of neutral sphingomyelinase 2 (nSMase2), an enzyme generating ceramide and critical for EV secretion, improved AD pathology, but this was not the case for female mice [13]. Therefore, we hypothesized that in female AD mice, other ceramide-generating enzymes compensated for inhibited or deficient nSMase2, leading to the production of EVs that intensified Aβ-mediated mitochondrial damage and neurotoxicity. To identify the enzyme responsible for the sexual dimorphism in the neurotoxicity of astrocyte-derived EVs, we compared the protein and ceramide content of EVs from male and female 5xFAD brains and determined their effect on mitochondrial function and cytotoxicity in neuronal cells.

We report that both wild type (WT) and 5xFAD females present more CD9(+) EVs compared to their male counterparts. In these EV populations, there is specific enrichment of Aβ, ceramide, and acid sphingomyelinase (ASM). ASM generates ceramide in lysosomes and the plasma membrane via the hydrolysis of sphingomyelin (SM) [14]. In the context of AD, the expression levels of ASM as well as its enzymatic activity are abnormally high [15,16]. ASM and Aβ are colocalized in female CD9(+) EVs at a single vesicle level. ASM activity was significantly higher in female EVs compared to males, both in WT and 5xFAD. In addition, our ExoView analysis indicated that CD9(+) EVs were of astrocytic origin and not from neurons. We confirmed the function of ASM in EV formation and Aβ binding using co-labeling and proximity ligation assays, showing that ASM inhibitors prevented complex formation between Aβ and ceramide in primary cultured astrocytes. Female EVs showed significantly greater mitotoxic effects, as quantified by Seahorse analysis, as well as increased cytotoxicity. Our study provides a rigorous and reproducible method for isolating small EVs (sEVs) as characterized by their size. By utilizing this method, we were able to demonstrate a sexual dimorphism in EVs derived from WT and 5xFAD mice. Specifically, we identified a unique subpopulation of sEVs that are enriched with ceramide, generated by ASM, and bind to Aβ, thus enhancing its neurotoxicity in the female brain. This finding suggests that sEVs and ASM could serve as novel diagnostic markers and therapeutic targets in AD.

## 2. Materials and Methods

### 2.1. Animals

C57BL/6 wild type (WT) and 5xFAD mice were bred in-house. Food and water were provided ad libitum, and animals were housed under a 12 h light/dark cycle in ventilated cages. Experimental groups were matched by sex and genotype (male and female, WT and 5xFAD). Intracardial profusion with ice-cold PBS was performed prior to brain collection to remove EVs from the brain’s circulation. Next, the brain was removed, washed with PBS, and sliced sagittally before carrying out the EV isolation protocol. In some experiments, a small portion of the cortex was collected for Western blotting. All experiments using mice to generate primary cell cultures for drug testing were carried out according to the Animal Use Protocol approved by the Institutional Animal Care and Use Committee at the University of Kentucky. At the end of the experiment, mice were terminally anesthetized.

### 2.2. Brain EVs Isolation

To cultivate EVs from mouse brains, nine- to twelve-month-old mice were anesthetized using isoflurane inhalation in a chamber. Whole-body perfusion with ice-cold PBS was performed to remove blood-derived EVs from the brain’s circulation. After careful separation of the brains, the olfactory bulb and cerebellum were removed, and the forebrains were washed in cold PBS and cut into eight sagittal slices using a sterile scalpel in a Petri dish. Brain tissues were transferred to Miltenyi Biotec C tubes supplemented with enzymatic dissociation buffer (1900 µL of buffer Z/50 µL enzyme P + 20 µL of buffer Y/10 µL enzyme A per brain, following the manufacturer’s instructions). Tightly closed C tubes were then attached upside down on the sleeves of a gentleMACS Octo dissociator with heaters, and the program 37C_ABDK_01 was used to dissociate brain tissues. Brain homogenates were applied to 70 μm MACS SmartStrainer (Miltenyi Biotec, Bergisch Gladbach, Germany), and EVs and cells were flushed out with 10 mL of cold Dulbecco’s PBS. The flow-throughs were centrifuged at 300× *g* for 10 min at 4 °C and cell pellets were discarded or used for other experiments. Each flow-through was transferred to a fresh 15 mL conical tube and supplemented with Halt™ Protease and Phosphatase Inhibitor Cocktail (Thermofisher Scientific, Waltham, MA, USA) at a 1:100 dilution, followed by centrifugation at 2000× *g* and 10,000× *g* for 10 min and 40 min, respectively. Supernatants were then passed through a 0.45 μm filter before following the ExoEasy Maxi kit (Qiagen, Germantown, MD, USA) or Optiprep™ isolation protocol following the manufacturer’s instructions. Briefly, to create a discontinuous gradient of iodixanol diluted in 0.25 M sucrose, we layered 3 mL each of 40%, 20%, and 10% iodixanol and 2.3 mL of 5% iodixanol. Samples of EVs obtained from 100,000× *g* (300–350 µL) were placed on top of the 5% portion and centrifuged at 100,000× *g* for 18 h at 4 °C using an SW41 swing-out rotor (Beckman Coulter, Brea, CA, USA). We collected 12 fractions (1 mL each) from the top and the protein content was measured in each fraction using the Thermo Scientific Pierce BCA protein assay kit according to the manufacturer’s instructions. Analysis of OptiPrep™ density gradient fractions indicated that the sEVs marker Alix1 was predominantly distributed to the fraction at 1.11 g/mL (fraction 7). Therefore, we used this fraction for subsequent analyses. For the ExoEasy protocol, the supernatant from the 10,000× *g* centrifugation was passed through a 0.45 μm filter to remove larger particles and aggregates. Next, a mixture of Exoeasy binding buffer (XBP) and the filtered sample (in a 1:1 ratio) was added to an Exoeasy spin column. The column was centrifuged at 500× *g* for 1 min, and the resulting flow-through was discarded. The spin columns were returned to the same tube, and 10 mL of Exoeasy washing buffer (XWP) was added to the columns. The columns were then centrifuged at 500× *g* for 5 min to remove residual buffer, and the resulting flow-through and tubes were discarded. The spin columns were transferred to fresh collection tubes. Finally, 400–1000 μL of elution buffer was used to elute the sample from the membrane. The eluate was collected by centrifuging at 4000× *g* for 5 min and transferred to fresh Eppendorf tubes.

### 2.3. Nanoparticle Tracking Analysis

A ZetaView PMX110 instrument (Particle Metrix, Meerbusch, Germany) was used for nanoparticle tracking analysis (NTA) and size/concentration quantification, as previously described [17]. Briefly, 2 mL of EVs suspended in PBS with the appropriate dilution were injected into the ZetaView cell. NTA measurements were obtained at 11 positions, with two cycles at each position. During acquisition, the temperature was set to 23 °C, with a camera sensitivity of 82, 30 frames/s, and a shutter speed of 150. Commercial 100 nm polystyrene beads (Particle Metrix) were used for instrument calibration. The Zeta potential was also measured using the ZetaView PMX110 instrument.

### 2.4. Immunoblot Analysis

Equal numbers of brain-derived EVs and the corresponding brain tissue lysate (normalized to protein concentration) were solubilized with 5 × Laemmli sample buffer (10% SDS, 1 mg/mL bromophenol blue, 250 mM Tris pH 6.8, 5% β-mercaptoethanol, 0.5 M DTT, 50% glycerol), followed by heating to 95 °C for 8 min. Protein separation was performed using pre-cast gels 4%–20% (mini-PROTEAN TGX, Biorad, Hercules, CA, USA) or SDS-PAGE gels (8% or 10%) and transferred to nitrocellulose membranes. Nonspecific binding was inhibited by blocking with 5% NFDM (Blotting-grade blocker, Biorad) for 1 h. Blots were then probed with primary antibodies overnight at 4 °C and incubated with an HRP-conjugated secondary antibody. Either SuperSignal™ West Femto Substrate (ThermoFisher, Waltham, MA, USA) or Clarity Western ECL Substrate (Biorad) were used for membrane development, and images were acquired on a ChemiDoc imaging system (Biorad) or Azure 600 system (Azure Biosystems, Dublin, CA, USA). Primary antibodies used were CD9, CD63 (ExoAb Antibody Kit, Systems Biociences, Palo Alto, CA, USA) at a dilution of 1:1000, GM130 (sc-55591, Santa Cruz Biotechnology, Dallas, TX, USA) at 1:200 dilution, ASM at 1:500 dilution (14609-1-AP, Proteintech), Flotillin 1 (15571-1-AP, Proteintech, Rosemont, IL, USA), Calnexin (sc-46669, Santa Cruz) at 1:200, and Alix (sc-53540, Santa Cruz) at 1:100.

### 2.5. ExoView

Equal numbers of brain-derived EVs were diluted in 1× incubation buffer and loaded on pre-scanned ExoView tetraspanin chips, placed in a sealed 24-well plate overnight at room temperature. The chips contained spots printed with anti-CD81 or anti-CD9 antibodies or mouse IgG1κ matching isotype antibodies, used as a control for non-specific EV binding (NanoView Bioscience, Brighton, MA, USA, EV-TETRA-MI). Chips were then moved to an automated ExoView^®^ CW100 Chip Washer using the tetraspanin program. The following antibodies were used to label EVs: anti-ceramide (produced in lab [18]) and anti-ASM (Proteintech–14,609) conjugated to Alexa Fluor 647 using the Zenon™ Rabbit IgG Labeling Kit (ThermoFisher), anti-CD81 conjugated to Alexa Fluor 555 (NanoView Bioscience, EV-mCD81-A-555) and anti-GFAP (Dako-Z0334) conjugated to Alexa Fluor 546 using the Zenon™ Rabbit IgG Labeling Kit (ThermoFisher), anti-beta amyloid 1–42 conjugated to Alexa Fluor 555 (Bioss Antibodies, Woburn, MA, USA—bs-0076R), anti-LCAM1 (Novus Biologicals, Englewood, CO—FAB5674X) conjugated to Alexa Fluor 532, anti-CD9 (NanoView Bioscience, EV-mCD9-A-488), and anti-CD63 (NanoView Bioscience, EV-mCD81-A-647). All antibodies were diluted in blocking solution at a concentration of 0.5–1.0 mg/mL. After washing and drying, chips were then imaged with the ExoView R100 reader using the ExoScan 3.0 acquisition software. Images acquired were analyzed using ExoViewer 3.0 software.

### 2.6. ASM Activity Assay

To assess the activity of acid sphingomyelinase (ASM) in sEVs, we performed an ASM assay on freshly isolated sEVs using a commercial kit (Echelon Biosciences, Salt Lake City, UT, USA, K-3200) following the manufacturer’s instructions. Fresh sEVs were isolated from the brains of male and female mice, and then sonicated in an ice-cold lysis buffer containing 150 mM NaCl, 50 mM Tris pH 7.4, 0.6% Triton X-100, and the protease inhibitor cocktail Halt™ (Thermo Fisher, PI78430). The resulting preparations were centrifuged at 10,000× *g* for 5 min, and roughly 2 × 10^11^ particles (10 μg protein) were used to measure ASM activity.

### 2.7. Cytotoxicity Assay

Neuro-2a (N2a) (ATCC-CCL-131™) cells were seeded at 1 × 10^4^ cells/well density on 96-well plates and allowed to grow at 37 °C and 5% CO_2_ atmosphere in DMEM media supplemented with 1% penicillin–streptomycin and 10% FBS (HyClone, GE Healthcare, Chicago, IL, USA). Cells were gradually deprived of serum for two days to differentiate N2a cells into the neuronal phenotype. After differentiation and reaching adequate confluency, cells were treated with an equal number of EVs (1 × 10^4^ EVs/cell) to determine the cytotoxicity of male and female brain-derived EVs. LDH release was detected using the CyQUANT™ LDH Cytotoxicity Assay (Thermo Fisher Scientific, Waltham, MA, USA, C20300) according to the manufacturer’s protocol.

### 2.8. Preparation and Treatment of Primary Astrocytes Cultures

P0-P1 (day of birth or next day) C57BL/6 wild-type mouse brains were used to isolate primary glial cells. Brains were dissociated in PBS containing 0.1 M glucose, then the homogenate was passed through a 40 µm filter and plated in T-25 flasks in DMEM (Life Technologies, Grand Island, NY, USA) supplemented with 1% penicillin/streptomycin solution and 10% fetal bovine serum at 37 °C in a humidified atmosphere containing 5% CO_2_. Cells were allowed to grow for 7 days, then adherent cells were passed to 24-well plates containing uncoated glass coverslips and cultured in DMEM (Life Technologies, Grand Island, NY, USA). Astrocytes were transfected with CD9 turboGFP-tagged plasmid (Origene-RG202000) using Lipofectamine™ 3000 Transfection Reagent (ThermoFisher- L3000008) according to the manufacturer’s protocol. The following day, cells were treated with 100 nM HiLyte™ Fluor 555 labeled Aβ that had been incubated overnight at 4 °C to form oligomers. After 24 h, cells were processed for immunocytochemistry. Briefly, the cells were washed three times with PBS before being fixed with a solution of 4% p-formaldehyde containing 0.5% glutaraldehyde in PBS for 15 min at room temperature. Permeabilization was achieved by incubating cells with 0.2% Triton X-100 in PBS for 5 min at room temperature. To prevent non-specific binding, cells were blocked with 3% ovalbumin/PBS for 1 h at 37 °C. Anti-ASM primary antibody (14609-1-AP, Proteintech) was then added at 1:500 dilution, and cells were incubated at 4 °C overnight. The following day, cells were washed with PBS and incubated with Alexa Fluor 647-conjugated donkey anti-rabbit IgG (Jackson ImmunoResearch Laboratories, West Grove, PA, USA) secondary antibody, which was diluted to 1:300 in 0.1% ovalbumin/PBS, for 2 h at 37 °C. The cover slips were mounted using Fluoroshield supplemented with DAPI (Sigma-Aldrich, St. Louis, MO, USA) to visualize the nuclei. Finally, the images were processed using Nikon NIS-Elements software that was equipped with a 3D deconvolution program.

### 2.9. Seahorse Assay

The bioenergetic/metabolic profile on N2a cells was assessed using a XF Cell Mito Stress Assay performed in a Seahorse XFe96/XF96 analyzer (Seahorse Bioscience/Agilent Technologies, Santa Clara, CA, USA). Cells were seeded at an initial 10^4^ cells/well in a XF microplate (Agilent) and allowed to grow in 5% FBS-supplemented media for 24 h prior to being deprived of serum and challenged with 10^4^ EVs/cell for 18 h.

### 2.10. Transmission Electron Microscopy (TEM)

Brain-derived EVs were fixed in a 2% PFA solution, and 5 µL of the fixed EVs were adsorbed on a hydrophilic glow-discharged FCF-200-Cu grid. Excess liquid was removed using filter paper, and the grids were placed on drops of water to remove any salts or phosphate. The grids were then stained with 1% uranyl acetate for 1 min, followed by three washing steps using distilled water droplets. The grids were air-dried and stored until imaging. Images of the EVs were acquired using a Thermo Scientific™ Talos™ F200X transmission electron microscope operating at a 200 kV accelerating voltage in scanning transmission electron microscopy (STEM) mode. To maximize contrast and minimize beam damage, we used low beam current and long dwell times (50 μs).

### 2.11. Proximity Ligation Assay

N2a cells were cultured and subjected to the same treatment as previously outlined in the cytotoxicity protocol. To prevent non-specific binding, the cells were treated with Duolink PLA blocking solution (Sigma-Aldrich) at 37 °C for 1 h. Primary antibodies, including anti-Aβ mouse IgG (1:500 4G8, Biolegends, San Diego, CA, USA, SIG-39220) and anti-ceramide rabbit IgG (produced in the lab), were used. Aβ treatments were performed at a final concentration of 1 µM Aβ1-42 after 2 h of preincubation with fluoxetine at 5 µM. For secondary PLA probes, anti-mouse MINUS affinity-purified donkey anti-mouse IgG (H  +  L) and anti-rabbit PLUS affinity-purified donkey anti-rabbit IgG (H  +  L) were diluted in antibody diluent buffer at a ratio of 1:5, and the samples were incubated at 37 °C for 1 h. The ligation and amplification steps were then performed in accordance with the manufacturer’s protocol (Duolink, Sigma-Aldrich, St. Louis, MO, USA). To visualize the nuclei, cover slips were mounted using Fluoroshield supplemented with DAPI (Sigma-Aldrich). ImageJ software (https://imagej.nih.gov/ij/, accessed on 1 July 2022) was used to analyze the images. The images were separated into two channels (DAPI and TRITC) to evaluate nuclear staining (DAPI) and the PLA dots (TRITC) separately. The threshold was adjusted to identify the nuclei and allow for binary conversion (black and white), and the morphological function was used to separate touching nuclei. The nuclei were counted and added to the region of interest (ROI), where the appropriate minimum and maximum pixel area sizes were set. In the other channel, the number of PLA dots (PLA signals) in each cell was calculated using the “Measure” command from the ROI manager, with single point as the output type.

### 2.12. Mass Spectrometric (Proteomic) Analysis of EV Proteins

The preparation of sEVs samples for peptide digestion and mass spectrometry was performed by lyophilizing the samples overnight and then resuspending them in a freshly made solution of 50 mM ammonium bicarbonate buffer containing 0.1% (*w*/*v*) RapiGest SF Surfactant (Waters) and 10 mM dithiothreitol. The mixture was heated to 60 °C for 30 min to reduce the disulfide bonds, followed by alkylation with iodoacetamide for 30 min in the dark. Subsequently, trypsin was added and allowed to digest the samples for 16 h at 37 °C. The reaction was stopped by adding trifluoroacetic acid to a final concentration of 0.1% (*v*/*v*), and the detergent was cleaved by incubating the samples at 37 °C for 40 min. After centrifugation at 15,000× *g* for 5 min, the supernatants were collected and transferred into sample vials for LC-MS analysis.

LC-MS analysis was performed using an Orbitrap Fusion tribrid mass spectrometer (Thermo Scientific, New York, NY, USA) connected to an Ultimate 3000 nano-UPLC system (Thermo Scientific), following the protocol described in our previous publications. Briefly, the peptide samples were trapped in a Pepmap100 C18 peptide trap, washed, and then separated on a Pepman 100 RSLC C18 column using a gradient of acetonitrile with 0.1% formic acid. The LC-MS/MS analysis was performed in positive mode with data-dependent acquisition using the Orbitrap MS analyzer for precursor scans and the ion-trap MS analyzer for MS/MS scans. Collision-induced dissociation was used to fragment the precursor peptides. The raw MS and MS/MS spectra were processed using the Proteome Discoverer software by Thermo Scientific (v1.4) and searched against the Uniprot human database using the SequestHT search algorithm. The Percolator PSM validator algorithm was used to validate the peptide spectrum matching and estimate the false discovery rate to be <1% (q-value) (https://www.nature.com/articles/nmeth1113, accessed on 12 April 2022).

For normalization, statistical analysis, and pathway analysis of genetic and sex-specific proteins, the peptide spectrum match (PSM) count for each identified protein in the LC-MS/MS search results was used as a semi-quantitative measure for protein expression level. The PSM count for each protein in a specific sample was first normalized using the sum of the PSM counts for all proteins in that sample. Then, the mean PSM count for the three replicates in each group was calculated for each protein and further used for statistical analysis. Protein content was compared between the different biological groups (sEVs). The EdgeR R package was used to perform trimmed mean normalization (TMM), and then the difference in protein expression between the groups was analyzed. Proteins up-regulated or down-regulated with a *p*-value cutoff of 0.05 were considered differentially expressed for further analyses. Gene Ontology pathway analyses were conducted using the Database for Annotation, Visualization, and Integrated Discovery (DAVID) and FunRich on differentially expressed protein genes. Uniprot Knowledgebase (UniProtKB) protein descriptions and gene products were imported into DAVID and FunRich for statistical analyses and GO term annotation based on integrated biological, molecular, and cellular pathways of the differentially expressed proteins.

### 2.13. Sphingolipid Analysis

The quantification of sphingolipids in brain-derived small extracellular vesicles (sEVs) was carried out by the lipidomics core facility at the Medical University of South Carolina, Charleston, SC, under the direction of Dr. Besim Ogretmen, as described previously [19]. The sphingolipid analysis included the determination of various species, such as sphingosine, sphinganine, sphingosine-1-phosphate, sphinganine-1-phosphate, and Cer (with N-acyl chain lengths ranging from C14:0 to C26:0). The resulting sphingolipid levels were normalized to the number of sEVs.

### 2.14. Statistical Analysis

GraphPad Prism (Version 9) was used to analyze data and prepare figures. The statistical analysis was conducted using either Student’s t-test for independent means or two-way ANOVA, followed by post hoc analysis using Tukey’s multiple comparisons test as appropriate. The significance level was set at *p* < 0.05. The mean ± SEM is reported for the data, and *n* represents the number of separate replicates. When the sample size was small, the conditional normality of the data was assessed by looking at a plot of the residuals from the analysis (a q–q plot or histogram).

## 3. Results

### 3.1. Characterization of Brain-Derived EVs

**Figure 1:**
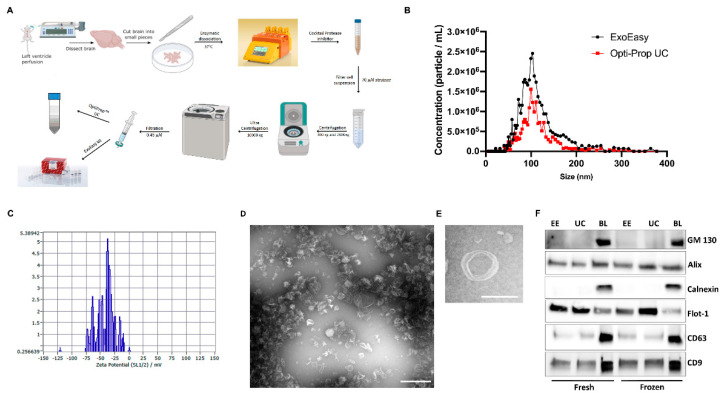
Isolation and characterization of brain-derived sEVs. (**A**) Schematic summary of the sEV isolation protocol from brain tissue. (**B**) Nanoparticle tracking analysis (NTA) showing the relative size distribution of sEVs using ExoEasy (black) or OptiPrep™ (Iodixanol) density gradient ultracentrifugation (red). (**C**) Representative Zeta potential measurement of brain-derived EVs. (**D**,**E**) TEM micrographs of uranyl acetate-stained sEVs using ExoEasy (**D**) and OptiPrep™ (Iodixanol) density gradient ultracentrifugation (**E**) showing cup-shaped morphology and heterogeneous sizes; the scale bar represents 500 nm (**D**) or 100 nm (**E**). (**F**) Western blot analysis of brain-derived sEVs. Equal numbers of ExoEasy (EE) or OptiPrep™ (Iodixanol) density gradient (UC) sEVs and the corresponding protein concentration from brain lysate (BL) were loaded. Proteins were separated by gel electrophoresis, and immunoblotting was carried out using antibodies to calnexin, GM 130, Flotillin-1, Alix1, CD9, and CD63. Immunoblots are representative of at least three independent experiments. See Supplementary Figure S1 for higher resolution images.

The biggest challenge in extracting EVs from brain tissue is preserving cell integrity during homogenization, as it can result in the release of intracellular vesicles, myelin debris, and fragments of cellular organelles. To overcome this, we adapted neuronal cell isolation techniques from Miltenyi Biotec to clear the extracellular space in brain tissue and enable access to and separation of EVs from the extracellular matrix. After digestion, the cells were pelleted by low-speed centrifugation at 300× *g*, and the remaining supernatant was transferred to fresh tubes for further centrifugation to remove tissue debris and larger EVs such as microvesicles. The purified supernatant was then passed through a 0.45 µm filter and subjected to either membrane affinity-based or iodixanol gradient-based EVs isolation (Figure 1A). The vesicles were rigorously characterized using techniques such as nanoparticle tracking analysis (NTA), transmission electron microscopy (TEM), immunoblotting, protein profile analysis, and ExoView analysis. To compare the distribution of vesicle sizes for the two methods, we used NTA, which determines the particle size distribution of samples in liquid suspension based on the properties of Brownian motion and light scattering.

The number of EVs recovered with the ExoEasy method was significantly higher than that of OptiPrep, and the average mean diameter of ExoEasy EVs was larger (105.2 ± 6.3 nm and 117.7 ± 14.2 nm, respectively) (Figure 1B). This was consistent with previous studies reporting that gradient ultracentrifugation techniques can compromise the yield of vesicles compared to other isolation methods [20]. The Zeta potential of the isolated EVs was within normal values, indicating colloidal stability that was not influenced by bioconjugation, surface chemistry, or the separation method used (Figure 1C). The size and morphology of the recovered particles were confirmed using TEM, which showed that both ExoEasy and OptiPrep techniques yield a heterogeneous population of vesicles between 50 nm and 200 nm in diameter (Figure 1E,F).

To evaluate the relative enrichment of cell-to-vesicle-associated proteins and compare the ExoEasy and OptiPrep methods under fresh and frozen conditions, forebrain homogenates with equal total protein content from EV preparations were separated by gel electrophoresis and immunoblotted for proteins commonly found in small EVs (sEVs). Since our purification method does not distinguish between EVs from the multivesicular endosome or the plasma membrane, the term “sEVs” is not restricted to exosomes but includes smaller microvesicles or ectosomes as well. There was no significant difference in the enrichment of sEV marker proteins, such as the tetraspanins CD81 and CD63, flotillin-1, and Alix, between ExoEasy and OptiPrep EVs in both fresh and frozen conditions. These proteins are typically localized to the sEV population and differentiate them from other types of vesicles [21]. In addition, we tested for contamination with membrane vesicles, typically resulting from tissue disintegration, since none of the current isolation techniques can separate them from EVs. The enrichment of markers for cellular organelles, such as the Golgi protein GM130 and the endoplasmic reticulum marker calnexin, in the forebrain homogenates but not in the EVs confirms that our isolation method minimized tissue disintegration and that the EV preparations were pure and free from cellular organelles and debris.

**Figure 2:**
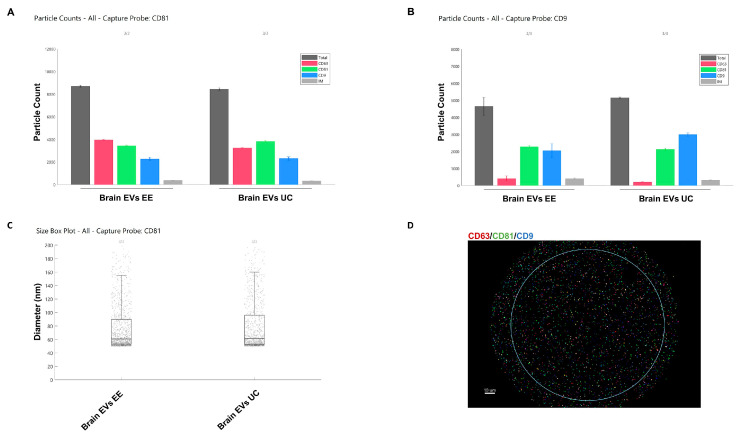
ExoView analysis of brain-derived sEVs. ExoEasy and OptiPrep-UC sEV from a pool of WT male and female samples were captured on the surface of tetraspanin chips printed with antibodies against CD81, CD9, and isotype probes at defined spots. sEVs bound to the anti-CD81 or anti-CD9 spots were then immunolabeled with AF488-conjugated anti-CD9 (blue), AF555-conjugated anti-CD81 (green), and AF647-conjugated anti-CD63 antibodies (red). (**A**,**B**) Representative bar charts of sEVs captured on the anti-CD81 and anti-CD9 spots, respectively. (**C**) Size distribution output of ExoView R100 showing no significant difference between the two methods. (**D**) Whole sEV populations were detected at the single-vesicle level, showing either one channel labeling and colocalization between two or three channels. See Supplementary Figure S2 for higher resolution images.

Next, we used ExoView analysis, which is based on immunocapturing EVs on microarray chips printed with anti-tetraspanins CD9 (HI9a) and CD81 (JS-81) capture probes as well as mouse IgG1κ isotype probes as controls for non-specific binding. An equal number of EVs in each preparation was incubated on microarray chips overnight, which resulted in the capture of similar numbers when using the two purification methods. In addition, the tetraspanin profile was comparable between the two methods, with similar expression of CD9, CD81, and CD63 on the surface of EVs on both CD9 (HI9a) and CD81 (JS-81) capture probes (Figure 2A,B). Moreover, the size determination of EVs from both methods showed similarity in the mean diameter of EVs given the specific calibration of the ExoView instruments (Figure 2C). Colocalization analysis of the tetraspanins showed the presence of a heterogeneous pool of vesicles, as depicted by the presence of either one, two, or three of the tetraspanins on the surface of EVs (Figure 2D). After establishing the similarity between the two methods, we focused on using the ExoEasy protocol with programmed tissue homogenization to minimize human error and reduce the differences between experimenters, making the results more reproducible.

### 3.2. Proteomic Analysis Elucidates EVs Sexual Dimorphism

Sexual dimorphism is highly manifested in the brain [22]. It is well established that the female brain is different from the male brain, both anatomically and functionally [23]. These differences indicate that biological sex may have a substantial influence on human cognitive functions, including emotion, memory, and perception, as well as a predisposition to neurodegenerative diseases [22,24,25]. To that end, females are at least two-fold more susceptible to Alzheimer’s disease compared to age-matched males [26,27]. Since EVs, especially sEVs, have been reported to play a significant role in advancing the progression of many neurodegenerative diseases, including AD, we decided to examine the differences in protein cargo composition of WT and 5xFAD mice of both sexes [6,28].

**Figure 3:**
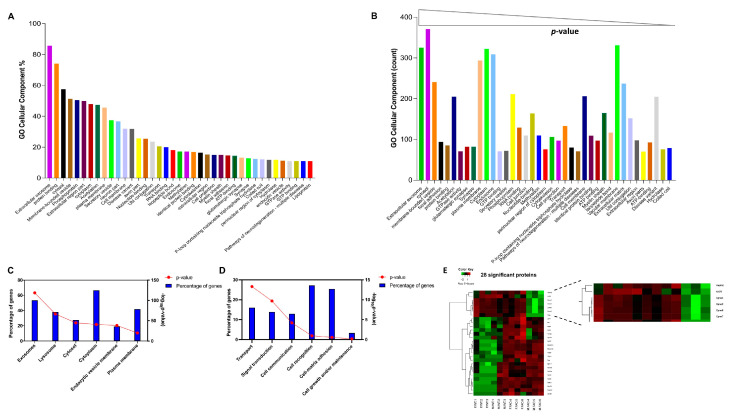
Cellular component GO terms and proteomic analysis of brain-derived sEVs. The proteins common to each biological sample (3 WT females, 3 5xFAD females, 3 WT males, and 3 5xFAD males) were grouped using gene ontology (GO) terms related to the cellular component analysis process using DAVID. (**A**) The graph shows the percentage of proteins identified by mass spectrometry that fall into the designated GO category relative to the total number of proteins in the category. (**B**) The proteins that fall into the designated GO category were ranked by significance. A modified Fisher’s exact *p*-value was used to demonstrate gene ontology; *p*-values less than 0.05 after Benjamini multiple test correction were considered enriched in the category. A count threshold of 5 and a default value of 0.05 for the enrichment score were used. (**C**) Functional analysis using FunRich showing the top cellular components. (**D**) Functional analysis showing the top biological functions of sEVs. (**E**) Heat map of the differential proteins. Protein expression values were log2-normalized, and cluster analysis was performed using Z-score protein intensities for the proteins with *p* < 0.05. Red indicates a high expression level; green indicates a low expression level. See Supplementary Figure S3 for higher resolution images.

The previous characterization showed that our method is able to purify functionally intact sEVs that are within the predicted size and shape and express common markers of sEVs. Therefore, we opted for proteomic analysis in order to get a better insight into the protein composition profile of the ExoEasy EVs and to unravel sex-specific protein expression in WT and 5xFAD brain-derived sEVs. Indeed, the proteins identified contained proteins implicated in the endosomal biogenesis pathway and trafficking of sEVs such as CHMP4B, VPS26, VPS50, PDC6IP, STX7, and SORT1. Gene ontology (GO) term analysis not only revealed that the term “extracellular exosomes” was among the top in terms of percentage and the first in terms of significance, but it also showed the absence of protein contamination from the Golgi, endoplasmic reticulum, or nuclei, which indicates that using this protocol leads to minimal co-isolation of cellular debris (Figure 3A,B). Moreover, using “FunRich,” which is an open-source functional enrichment and network analysis tool, we show that the top cellular components identified included identifiers for exosomes, lysosomes, and the endocytic vesicle membrane (Figure 3C) [29]. Functional analysis for the main biological processes revealed that transport, signal transduction, cell communication, and cell-matrix adhesion are among the top biological processes, all of which are considered typical for sEVs (Figure 3D).

Between all biological samples (3 WT females, 3 WT males, 3 5xFAD females, and 3 5xFAD males), there were 651 common proteins. Out of these common proteins, 28 were differentially expressed between the groups (Figure 3E). Indeed, the obvious candidate amyloid precursor protein (APP) level was significantly increased in the 5xFAD samples, both male and female. Myosin heavy chain (MYH11) was elevated in the 5xFAD samples as well, especially in females. MYH11 functions as a contractile protein, thus participating in cell movement and the transport of materials within and between cells. Interestingly, MYH11 has recently been reported to be differentially expressed in the female AD cortex, and variants within the gene are associated with dementia in females [30]. In addition, our analysis also indicated the downregulation of several proteins of the copines family in the male 5xFAD EVs, including Cpne2, Cpne4, Cpne7, and Cpne9. Together, these results suggest that our new method produces viable sEVs consistent with the experimental guidelines of the International Society of Extracellular Vesicles (ISEV). While proteomics clearly demonstrates the purity of sEVs and the presence of Aβ when isolated from 5XFAD brains, the functional significance of distinct proteins identified in sEVs from males and females remains to be investigated.

### 3.3. CD9 Is Differentially Expressed in Male and Female sEVs

**Figure 4:**
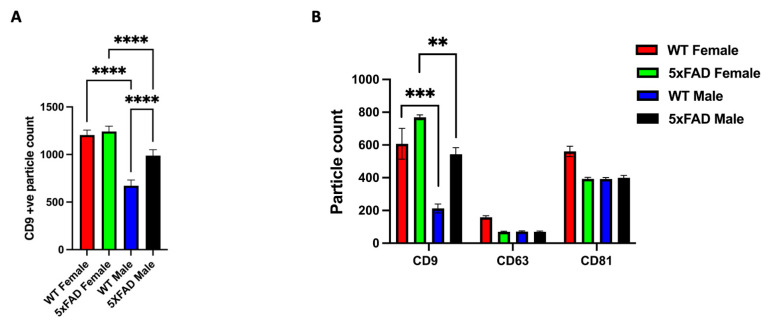
CD9 is differentially expressed in male and female brain-derived sEVs. (**A**) ExoView analysis of brain sEVs showing total EVs captured on anti-CD9 antibodies on tetraspanin chips. (**B**) Quantitative analysis for CD9 capture probe-bound EVs showing higher counts of CD9(+) vesicles in both WT and 5xFAD females compared to their male counterparts. The graphs are representative of at least three experiments where three biological samples were pooled for each group (total of *n* = 9 per group). Two-way ANOVA, Tukey’s test (** *p* < 0.01, *** *p* < 0.001, ****p < 0.0001). See Supplementary Figure S4 for higher resolution images.

**Figure 5:**
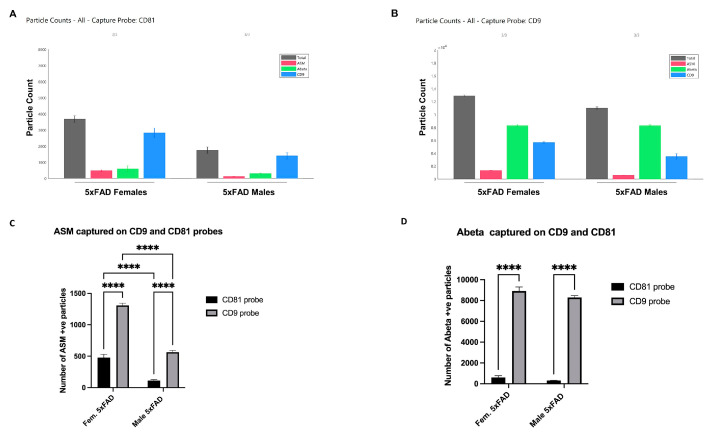
Specific enrichment of ASM in female CD9(+) sEVs. ExoView analysis showing higher counts of ASM (+) particles in female brain-derived sEVs compared to males (**A**,**B**). In addition, Aβ1-42 is significantly enriched on sEVs immunocaptured on the CD9 probe compared to the CD81 probe (**B**). (**C**) Raw data quantification of ASM (+) particles captured on both CD9 and CD81 probes; (**D**) raw data quantification of Aβ1-42(+) particles bound to CD9 and CD81 probes. Bar graphs represent average +/− SEM with *n* = 3/group. Two-way ANOVA followed by Tukey’s test (**** *p* < 0.0001). See Supplementary Figure S5 for higher resolution images.

Next, we examined the differences in male and female brain EVs at a single vesicle level using ExoView microarrays with anti-tetraspanins CD81 and CD9 printed on the chip surfaces. After incubating an equal number of EVs from each biological group (a pool of three), we found that both wild-type and 5xFAD female brain EVs contained a higher number of CD9(+) vesicles compared to their male counterparts, as indicated by the number of particles captured by the CD9 probe (Figure 4). However, we observed a similar expression of the other tetraspanins in all four groups (as shown in Figure 5). This suggests that male and female sEVs have distinct tetraspanin signatures, with CD9 being more prominent in female EVs.

Unlike the protein cargo composition, the tetraspanin composition could provide a deeper insight into the fundamental differences between male and female EV heterogeneity. According to previous studies, CD63 and CD81 are commonly known markers for sEVs (especially exosomes), while CD9 tends to be a marker for both microvesicles/ectosomes and sEVs [31,32]. This could imply that either female EVs express higher levels of CD9 or a portion of the isolated sEVs are small microvesicles/ectosomes that are CD9(+).

### 3.4. Specific Enrichment of ASM on Female CD9(+) EVs and Colocalization with Aβ

Based on previous studies showing that a subpopulation of CD9(+) EVs relies on ceramide generation by ASM [33], we immunocaptured sEVs on anti-CD9 and CD81 microarrays and co-labeled the vesicles with fluorescent anti-ASM antibodies. Consistent with the previous experiment, our results showed that female sEVs contained more CD9(+) vesicles compared to males, with similar results obtained when using both CD9 and CD81 probes (Figure 5A,B). Additionally, we found that vesicles bound to the CD9 probe had significantly more ASM compared to those captured on the CD81 probe. Furthermore, female sEVs from 5xFAD mice had significantly more ASM compared to male sEVs (Figure 5C). We also used a fluorescent antibody against Aβ1-42 and found no significant difference in its level, but similarly observed that Aβ1-42 was localized to CD9(+) vesicles (Figure 5D).

**Figure 6:**
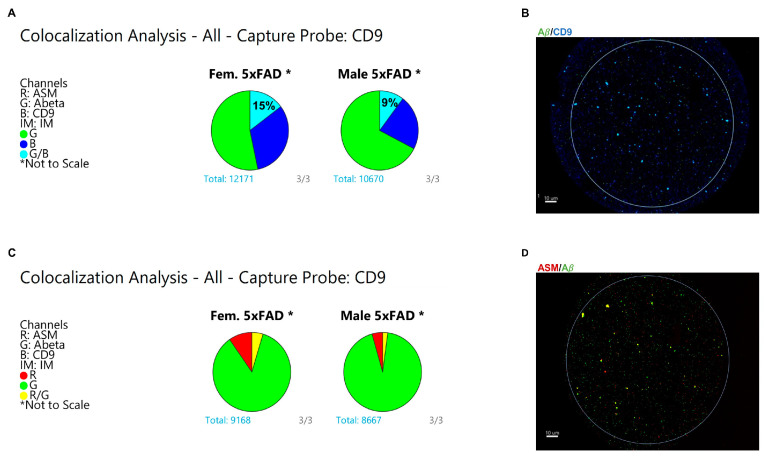
CD9(+) sEVs from female 5xFAD brains contain higher levels of ASM, which colocalizes with Aβ. (**A**) ExoView colocalization analysis of male and female 5xFAD brain-derived EVs captured on the CD9 probe showing increased colocalization between Aβ1-42 (green) and CD9 (blue) in female EVs compared to males. (**B**) Representative micrographs of female 5xFAD EVs bound to the anti-CD9 spot on the ExoView chip showing colocalized Aβ1-42 and CD9 channels. (**C**) ExoView colocalization analysis of male and female 5xFAD brain EVs captured on the CD9 probe showing a two-fold increase in the colocalization between Aβ1-42 (green) and ASM (red) in female EVs compared to males. (**D**) Representative micrographs of female 5xFAD CD9-captured EVs showing colocalized Aβ and CD9 channels. See Supplementary Figure S6 for higher resolution images.

To confirm that both ASM and Aβ1-42 were present on the same type of vesicles and not dispersed across different EV types, we conducted a colocalization analysis using the ExoView^®^ Analyzer software. Figure 6A,B show that in female 5xFAD brains, a larger proportion of CD9(+) sEVs is colocalized with Aβ1-42 than in male sEVs. Similarly, female 5xFAD brain sEVs showed a higher degree of colocalization between ASM and Aβ1-42 compared to males (Figure 6C,D). This suggests the presence of a distinct population of sEVs in female brain preparations that are CD9(+) and carry both ASM and Aβ as a cargo.

### 3.5. Astrocytes Are the Major Origin of CD9/ASM Vesicles in Female 5xFAD Brains

**Figure 7:**
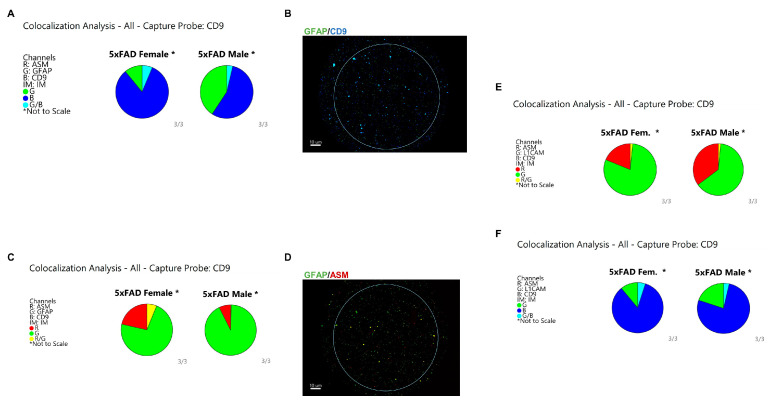
ExoView colocalization analysis shows that CD9/ASM (+) sEVs are derived from astrocytes. (**A**) Pie chart showing difference in colocalization between GFAP (AF534) and CD9 (AF488). (**B**) Representative spot image of the CD9 capture probe generated by ExoView showing CD9/GFAP colocalization in 5xFAD female sEVs. (**C**) Pie chart showing the specific colocalization between GFAP (AF534) and ASM (AF647) in 5xFAD female sEVs. (**D**) Representative spot image of the CD9 capture probe showing GFAP/ASM colocalization in female sEVs. (**E**) Colocalization analysis between ASM (AF647) and L1CAM (AF534) showing no difference between males and females; a similar observation was found between L1CAM (AF534) and CD9(AF488) in (**F**). See Supplementary Figure S7 for higher resolution images.

Next, we determined the cell type that secretes CD9(+) sEVs containing ASM and Aβ1-42 in the 5xFAD brain. While neurons are known to be the major producers of Aβ in the brain, previous studies, including our own work, have shown that astrocyte-derived EVs carry a higher amount of Aβ compared to neuron-derived EVs [12,34,35]. In order to investigate whether the cellular origin of Aβ-associated sEVs is sex-specific, ExoView analysis was conducted using fluorescent antibodies specific to glial fibrillary acidic protein (GFAP), which is a marker for astrocytes, and L1 cell adhesion molecule (L1CAM1), which is a marker for neurons. The colocalization charts showed that female sEVs have a higher degree of colocalization between GFAP and CD9 (Figure 7A,B). This is noteworthy because the data indicates that GFAP-positive sEVs are fewer in 5xFAD females than in males, yet the colocalization with CD9 is still significantly higher than in males (Figure 7A,B). Similar observations were made when examining the colocalization between GFAP and ASM, which showed that the colocalization between the two proteins almost exclusively occurred in females and not in males (Figure 7C,D). Interestingly, there was minimal colocalization between L1CAM1 and ASM, with no difference between male and female samples (Figure 7E). The CD9/L1CAM1 colocalization also showed no difference between males and females (Figure 7F). These data indicated that the CD9(+) sEVs carrying Aβ and ASM in female 5xFAD brains are mainly derived from astrocytes.

### 3.6. ASM Inhibitors Prevent Complex Formation between Ceramide and Aβ in Astrocytes

**Figure 8:**
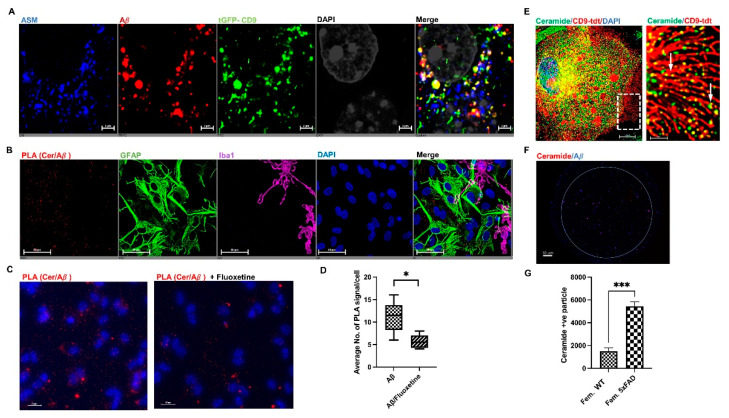
ASM mediates the formation of astrocyte derived CD9/Cer/Aβ (+) vesicles in female 5xFAD brains. (**A**) Representative fluorescence micrographs of primary cultured astrocytes transfected with tGFP-CD9 plasmid (green), treated with fluorescent Aβ1-42 (red), and immunolabeled for ASM (blue) showing colocalization between the three channels in the perinuclear area (merge). (**B**) Representative image of a mixed culture of astrocytes (GFAP, green) and microglia (Iba1, magenta) showing that PLA signals between ceramide/Aβ1-42 are predominantly found in astrocytes. (**C**) Representative image showing the effect of fluoxetine pretreatment on reducing the Cer/Aβ1-42 PLA signal count in astrocytes; (**D**) shows the quantification. (**E**) Colocalization of ceramide and tdTomato-CD9 in punctate staining suggesting secretion of ceramide-enriched sEVs. (**F**) Female sEVs immunocaptured on the ExoView CD9 probe showing the presence of ceramide and Aβ1-42. (**G**) Quantification of ceramide (+) sEVs showing that female sEVs contain a higher count of ceramide (+) sEVs compared to WT. Bar graphs represent average +/− SEM with *n* = 6/group for (**D**) and *n* = 3/group for (**G**), unpaired t-test (* *p* < *0*.05, *** *p* < 0.001). See Supplementary Figure S9 for higher resolution images.

**Figure 9:**
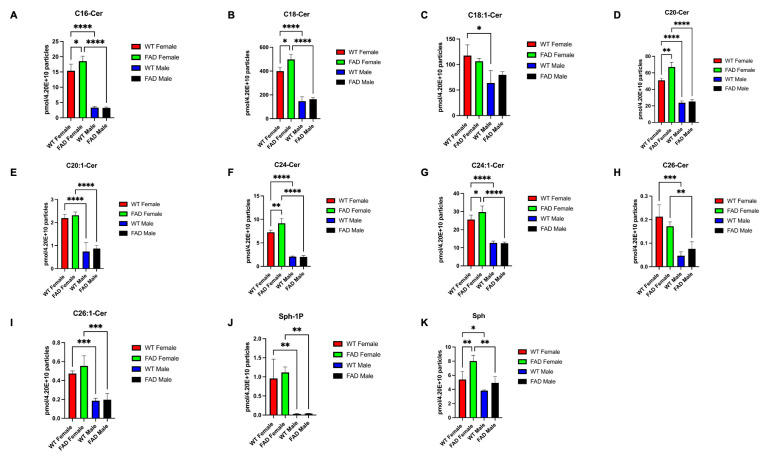
WT and 5xFAD female brain-derived sEVs express elevated levels of several ceramide species. (**A**–**K**) Quantification of C16:0, C18:0, C18:1, C20, C20:1, C24:0, C24:1, C26:0, and C26:1 ceramide, sphingosine, and sphingosine 1-phosphate by mass spectrometry of brain-derived sEVs. Lipid concentration was normalized to sEV count. The bar graph represents the average +/−SEM with each *n* = 3/group. Two-way ANOVA, Tukey’s test (* *p* < 0.05, ** *p* < 0.01, *** *p* < 0.001, **** *p* < 0.0001). See Supplementary Figure S10 for higher resolution images.

The colabeling of GFAP/CD9(+) sEVs for ASM and Aβ1-42 suggested that ASM generates ceramide, which leads to binding of Aβ to CD9(+) sEVs in astrocytes. To test if ASM colocalizes with CD9 and Aβ1-42, we transfected primary cultured astrocytes with Turbo-GFP and tdTomato-tagged CD9 plasmid, treated the cells with HiLyte™ Fluor 555-conjugated Aβ1-42, and immunolabeled for ASM. As shown in Figure 8A, we detected colocalization of ASM, Aβ1-42, and CD9 in perinuclear vesicles, suggesting they were of endolysosomal origin. Our group has previously reported that ceramide is required for the binding of Aβ to astrocyte-derived EVs. Therefore, we hypothesized that ASM is required to form a complex of Aβ1-42 with ceramide in astrocytes prior to the secretion of Aβ-associated sEVs. The complex formation was quantified by a proximity ligation assay (PLA) using antibodies against Aβ1-42 and ceramide in the absence or presence of ASM inhibitors. Our data using a mixed glial culture showed that the interaction between ceramide and Aβ1-42 is initiated predominantly in astrocytes (Figure 8B), and inhibiting ASM using the functional inhibitor fluoxetine significantly reduced the number of PLA signals indicative of preventing ceramide-Aβ1-42 complex formation (Figure 8C,D). The effect of ASM inhibition on ceramide-Aβ1-42 complex formation was confirmed using Arc39, a direct ASM inhibitor (Supplementary Figure S8). In addition, ceramide punctate staining colocalized with CD9 in proximity to the astrocyte plasma membrane, consistent with the secretion of CD9(+) ceramide-enriched sEVs (Figure 8E). The presence of ceramide and Aβ1-42 in or on the surface of female 5xFAD CD9(+) EVs was shown using ExoView utilizing antibodies against ceramide and Aβ1-42 on anti-CD9-captured vesicles, which contained significantly higher ceramide levels than control sEVs (Figure 8F,G). High levels of several ceramide species in female sEVs were confirmed using mass spectrometric (lipidomics) analysis. Figure 9A–K shows that the levels of C18-Cer as well as C16-Cer, C20-Cer, C24-Cer, and C24:1-Cer were significantly increased in female sEVs, suggesting that there is a specific mechanism in place that generates and/or sequesters ceramide to sEVs in the female brain. Moreover, ceramide enrichment in female sEVs could be attributed to the level and activity of ASM. In summary, our data indicated that ceramide was generated by ASM-mediated binding of Aβ to sEVs, which were then secreted from astrocytic processes, particularly in female 5xFAD brains.

### 3.7. Female sEVs Exert Increased Mitochondrial Bioenergetic Disturbance and Elevated Neurotoxicity

**Figure 10:**
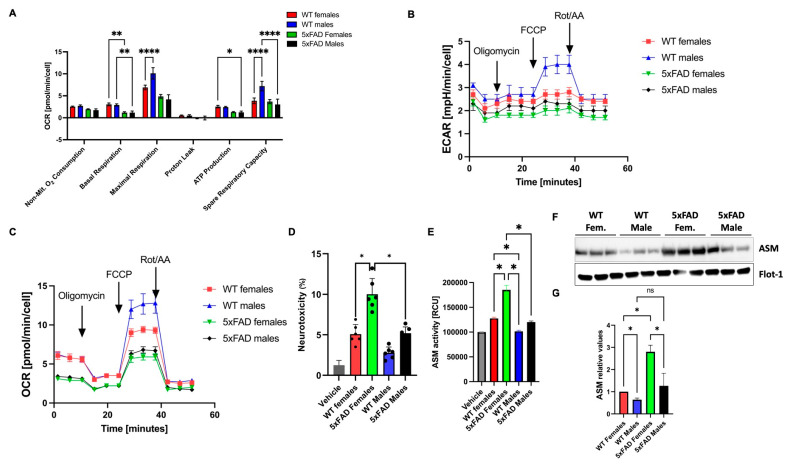
Female sEVs are more toxic than males. (**A**–**C**) Bioenergetics assessment of neuronal (N2a) cells incubated with WT and 5xFAD male and female brain-derived sEVs performed by a Seahorse Flux Analyzer using a Cell Mito Stress test. Lines and bar graphs represent average +/−SEM with each *n* = 8/group. Two-way ANOVA, Tukey’s test (* *p* < 0.05,. (**D**) LDH-release assay showing higher toxicity of 5xFAD female sEVs compared to the rest of the groups, *n* = 3/group. (**E**) ASM activity assay performed for freshly isolated brain-derived sEVs, *n* = 3/group. (**F**) Immunoblots showing increased ASM protein levels in WT and 5xFAD sEVs using Flot-1 for normalization, (**G**) shows the quantification of band intensities. Bar graphs represent average +/−SEM with each *n* = 3/group. Two-way ANOVA followed by Tukey’s test (* *p* < 0.05, ** *p* < 0.01, **** *p* < 0.0001). See Supplementary Figure S11 for higher resolution images.

Our previous research and that of others indicated that EVs taken up by neurons ultimately interact with and affect mitochondrial function [12,36,37], which could lead to mitochondrial fragmentation, disturbance of bioenergetics, or induction of apoptosis through cytochrome C release. Therefore, we tested whether brain sEVs exhibit sexual dimorphism in terms of mitotoxicity and neurotoxicity. Figure 10A–C shows that there was a significant genotype-specific decrease in both the oxygen consumption rate [OCR] and the extracellular acidification rate [ECAR], with both male and female sEVs affecting the two procedures negatively. Indeed, female 5xFAD sEVs induced more reductions in OCR and EACR than their male counterparts, indicating a more deleterious effect on mitochondrial respiration and glycolysis. Interestingly, WT female sEVs show more mitotoxic effects compared to males. In addition, when assessing the neurotoxicity of sEVs using the LDH assay, we found that female 5xFAD vesicles were significantly more neurotoxic than sEVs from male 5xFAD brains (Figure 10D). This could be due to the elevated ASM activity and expression, as well as a higher ceramide concentration in the female sEVs, which were transported into neurons and potentially increased the ceramide content inside neurons, which ultimately led to mitochondrial damage (Figure 10E–G).

## 4. Discussion

The term extracellular vesicle (EV) is a comprehensive descriptor for membrane-derived vesicles that are released by nearly all cell types and are present in all bodily fluids [38]. EVs contain a variety of lipids, metabolites, nucleic acids, and proteins that are involved in intercellular communication and cellular clearance processes [39,40]. Despite their ubiquity and potential importance, the precise composition and functions of EVs remain poorly understood due to the lack of reliable and reproducible separation protocols, particularly for samples from the central nervous system, such as animal and human brain tissues. Of interest to us are small extracellular vesicles (sEVs), including exosomes and small microvesicles and ectosomes, given the plethora of research implicating them in many neurodegenerative diseases.

To address these issues and gain a better understanding of sEVs, we describe the development of novel protocols for separating sEVs from unfixed frozen brain tissues of mice using gentle enzymatic dissociation followed by the affinity membrane capture method. Subsequently, we assessed the isolated sEVs through various techniques to examine their morphological, biophysical, and proteomic properties, including nanoparticle tracking analysis, transmission electron microscopy, and label-free mass spectrometry for protein profiling. Our results suggest that the isolated sEVs match the latest ASEMV guidelines in terms of size, shape, protein content, and the absence of cellular organelle contaminants.

In recent years, studies have brought forth the idea that EVs are not a homogenous pool of vesicles but rather heterogeneous [31,41]. It has been described that different cell types secrete sEVs with a distinct cargo composition specific to the donor cell [42]. Moreover, even from the same cell, the secreted sEVs contain different subpopulations that differ from each other, mostly via distinct tetraspanin enrichment and functionally significant cargo [43]. To that end, we compared sEVs from male and female WT and 5xFAD brain tissues.

Gender-specific differences in sEVs miRNA and protein cargo have been observed in various disease conditions, including ischemic heart disease (IHD) [44] and osteoarthritis (OA) [45,46]. These differences are mainly attributed to the differential up-regulation and down-regulation of proteins and miRNAs. In the brain, sexually dimorphic brain fatty acid composition has been reported in mice fed with low and high-fat diets [47]. Furthermore, neuroinflammation also seems to affect sEVs cargo in a sex-specific manner [48].

However, sexual dimorphism within sEVs has not been studied in the context of AD. Our proteomic analysis was able to delineate the difference between WT and 5xFAD mice and also between male and female sEVs. APP levels were significantly elevated in 5xFAD sEVs compared to WT. Interestingly, the male 5xFAD sEVs lacked many proteins when compared to the rest of the groups. Those proteins included Cpne2, Cpne7, and Cpne9, all of which belong to the copine family of proteins. Copines are a novel family of ubiquitous Ca (2+)-dependent, phospholipid-binding proteins that are highly conserved in a variety of eukaryotes [49]. For instance, Cpne7 regulates ciliogenesis during odontoblast differentiation, while CPNE2 plays a key role in regulating lysosomal-mitochondrial function involved in aging and disease [50,51]. Copines have also been shown to synergize with Annexin A1, another phospholipid-binding protein enriched in microvesicles, to form phosphatidylserine-enriched scaffolds, which may regulate membrane fusion and/or EV uptake [52]. Hence, further studies are needed to investigate the role of copines in female EVs, or the lack thereof, in male EVs from AD brains.

Furthermore, ExoView analysis revealed that the tetraspanin composition appears to have a sex-specific pattern, with CD9(+) sEVs being more abundant in female samples from WT and 5xFAD brains. This is particularly interesting because CD9(+) sEVs have been implicated in a variety of pathological conditions, including cancer and infectious diseases, suggesting that CD9 is not merely a surface tetraspanin for sEVs [53,54,55,56,57]. For instance, CD9(+) EVs in cancer have been shown to support tumor progression, invasion, and metastasis by transferring oncogenic proteins and miRNAs to recipient cells [58]. Furthermore, CD9(+) EVs have been detected in the plasma of cancer patients and have the potential to be used as diagnostic and prognostic biomarkers. In infectious diseases, CD9(+) sEVs have been shown to participate in the immune response to viral and bacterial infections. For example, CD9(+) sEVs derived from dendritic cells can activate T cells and augment the immune response to viral infections [58]. With respect to the membrane biophysical function of CD9 in EVs, one should notice that tetraspanins and ceramides are membrane curvature-inducing proteins and lipids, respectively [59,60,61,62]. This is consistent with the observation that microvesicles shed by outward budding of the plasma membrane are enriched with CD9 and require ceramide generated by ASM for secretion, particularly in astrocytes.

In addition to CD9, we found ASM to be enriched in female sEVs compared to males. Ceramide has been described as a major component of sEVs for many years, and the other ceramide-generating enzyme, nSMase-2, has been linked to the formation of exosomes through its role in facilitating the budding of intraluminal vesicles (ILVs) into late endosomes or the multivesicular endosome [60]. On the other hand, ASM is predominantly found in lysosomes and at the plasma membrane [63]. Studies have found that ASM is carried by cerebrospinal fluid (CSF)-derived extracellular vesicles in certain disease conditions, such as multiple sclerosis, with disease severity correlating with the number of ASM-enriched sEVs [64]. In our analysis, we report that ASM and Aβ colocalized on CD9(+) vesicles in female samples. This colocalization occurs mostly in EVs from astrocytes, which is in line with our previous research suggesting that astrocytes secrete sEVs that are enriched with ceramide and enhance the toxicity of Aβ when taken up by neurons [12]. However, given the fact that the neurons are the main source of Aβ, it is plausible that the neurons secrete a significant amount of sEVs that carry Aβ as their cargo. Therefore, further efforts are needed to delineate and even quantify the cell-specific contribution to the secretion of Aβ-bound sEVs and whether ASM plays a role in the incorporation of Aβ into sEVs in a distinct cell-type manner. It is important to note that while we used L1CAM to identify neuron-derived sEVs, it is also expressed by other cell types, including cancer cells. However, since our EVs are directly isolated from brain tissues, it is likely that L1CAM mainly corresponds to neurons. Our previous studies demonstrated that ceramide enrichment was critical for the neurotoxicity of Aβ-associated EVs. In the current study, we found complex formation between ceramide and Aβ in primary cultured astrocytes, which was significantly reduced using fluoxetine and Arc39, functional and direct ASM inhibitors, respectively [65]. This suggests that ASM facilitates the formation of ceramide-enriched female sEVs, which bind to Aβ and are then secreted and either taken up by neurons or deposited in the extracellular space, initiating plaque formation. This observation might explain why inhibiting nSMase-2 with the pharmacological inhibitor GW4869 reduces Aβ load and neurotoxicity only in male mice [13]. It is possible that the higher level of activity of ASM in females as shown in our experiment could act as an additional or complementary mechanism that leads to the secretion of CD9(+) sEVs from astrocytes that are enriched with ceramide and Aβ. Of note, while we used 5xFAD mice in this study to investigate the association of Aβ with sEVs, one should consider that the pathology mediated by sEVs may not account for the full spectrum of the disease, and in future studies, additional mouse models will be utilized to further delineate the sex-specific sEV composition and their subsequent effects. More specifically, to address limitations from using amyloidopathy mouse models such as 5xFAD, in future studies we will include models for tauopathy and neuroinflammation, as well as using our novel isolation method on human samples to test if similar sex-specific characteristics of EVs are found in human AD.

In summary, our study highlights the importance of sexual dimorphism in sEVs in the context of AD. Further research is needed to determine the role of copines and CD9(+) sEVs in AD pathogenesis, as well as the significance of ASM in sEV secretion and Aβ toxicity. Given this function of ASM, it is reasonable to suggest ASM as a drug target to decrease the ceramide levels critical for Aβ binding of sEVs and therefore effective in both sexes when aiming at ceramide metabolism in AD therapy.

## Data Availability

Data supporting reported results can be provided upon reasonable request.

## Supplementary Materials

The following supporting information can be downloaded at: https://www.mdpi.com/article/10.3390/cells12121623/s1, Figures S1–S7 and S9–S11: Higher resolution images for Figure 1, Figure 2, Figure 3, Figure 4, Figure 5, Figure 6, Figure 7, Figure 8, Figure 9 and Figure 10. Figure S8: ASM direct inhibitor Arc39 reduces the complex formation between ceramide and Aβ in astrocytes. Representative micrographs of epifluorescence showing the inhibitory effect of Arc39 on the interaction between ceramide and Aβ_1-42_. Primary cultured astrocytes were treated with 1 μM Aβ_1-42_ (green) with or without pretreatment with Arc39 at 40 μM. PLA dots represent ceramide (anti-ceramide rabbit IgG) and Aβ_1-42_ (anti-Aβ_1-42_ mouse IgG clone 4G8) interactions (red). Ceramide is visualized using a Alexa647-anti rabbit IgG secondary antibody (purple).
