# Supplementary figures and images for: Novel Isolation Method Reveals Sex-Specific Composition and Neurotoxicity of Small Extracellular Vesicles in a Mouse Model of Alzheimer’s Disease
Source: Cells. 2023 Jun 14;12(12):1623. doi: 10.3390/cells12121623 (PMC10297289; doi:10.3390/cells12121623)

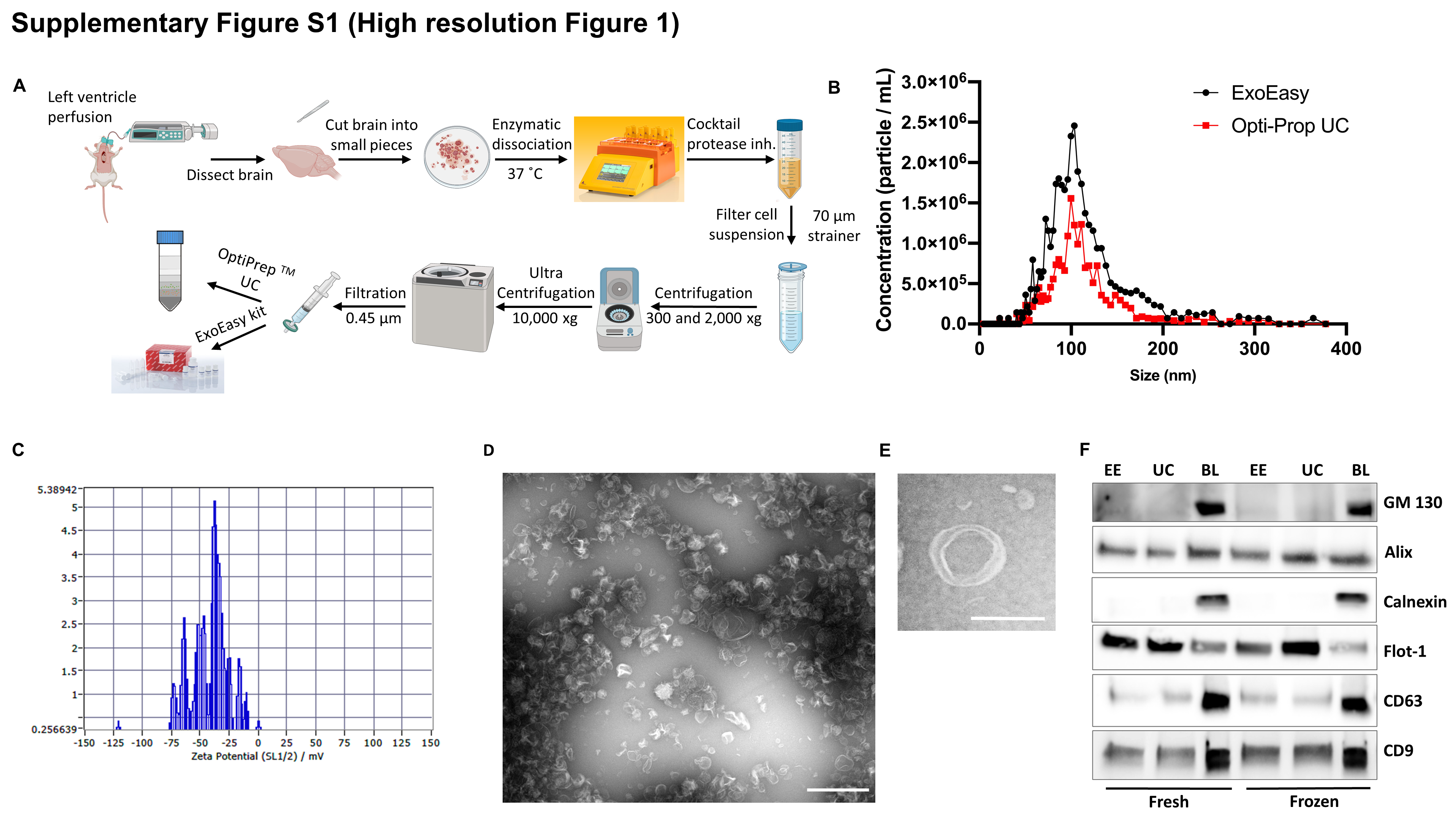

Supplement: Supplementary file 1 [file cells-12-01623-s001.zip › Figure S1.tif]

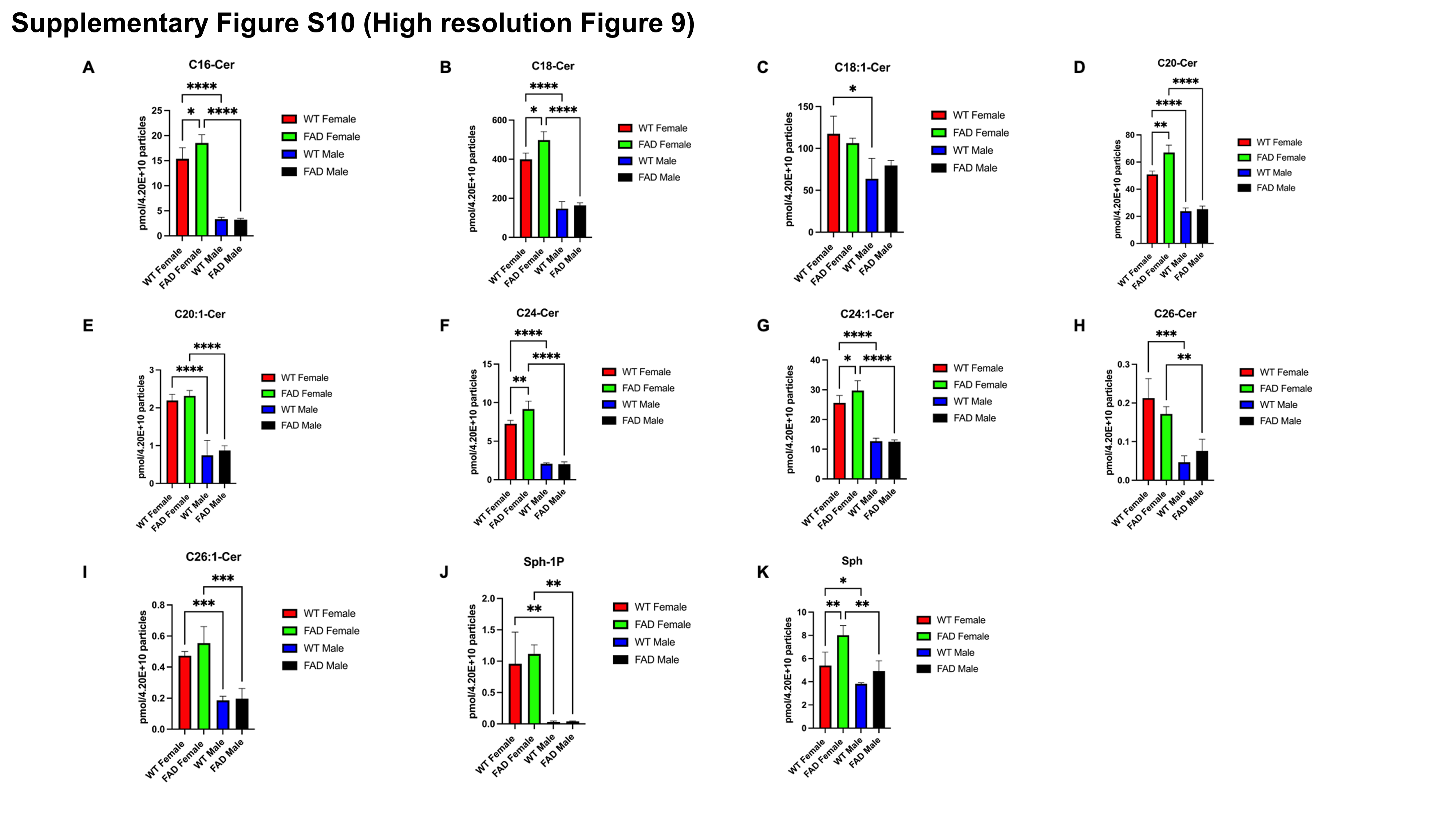

Supplement: Supplementary file 1 [file cells-12-01623-s001.zip › Figure S10.tif]

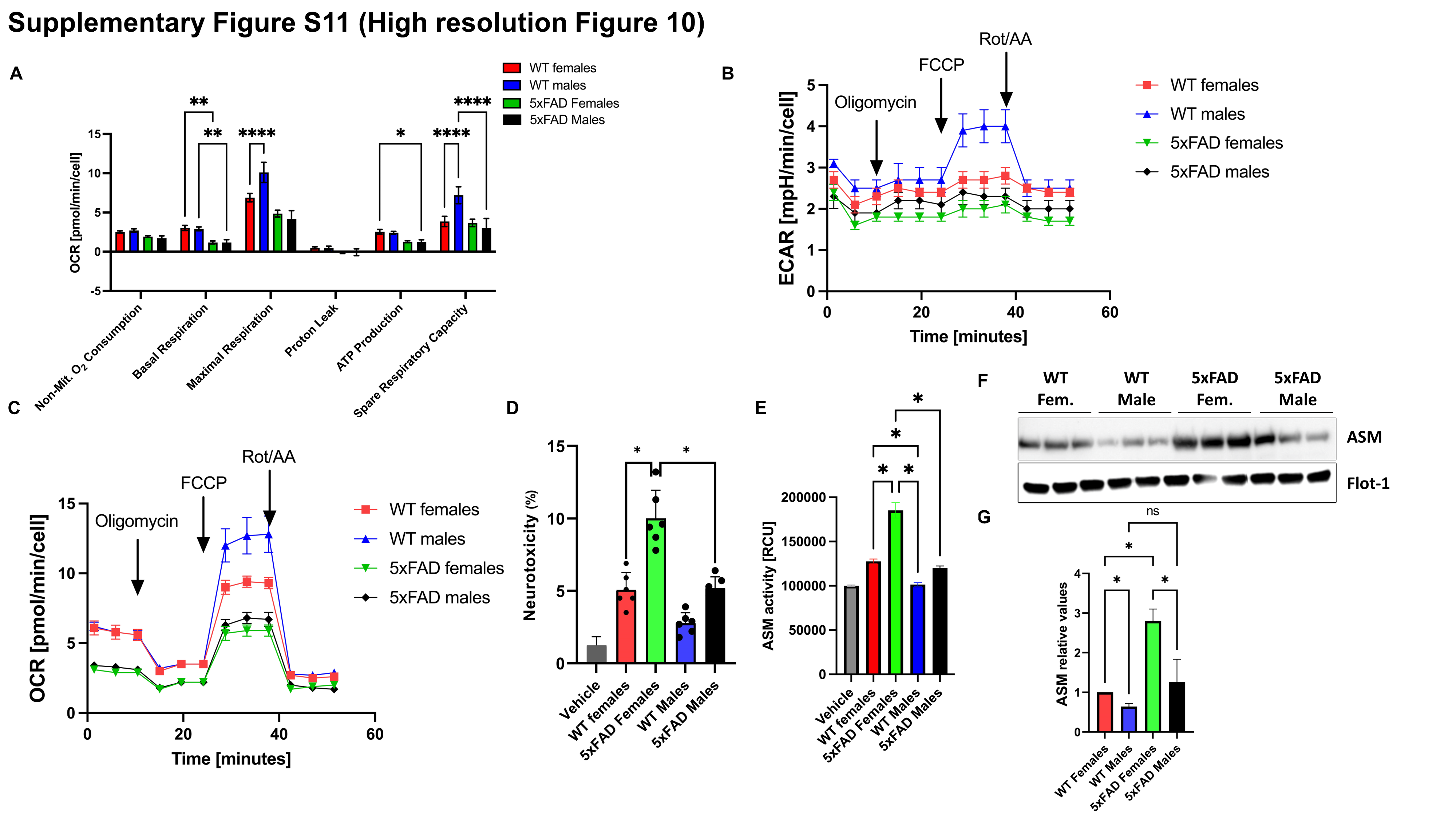

Supplement: Supplementary file 1 [file cells-12-01623-s001.zip › Figure S11.tif]

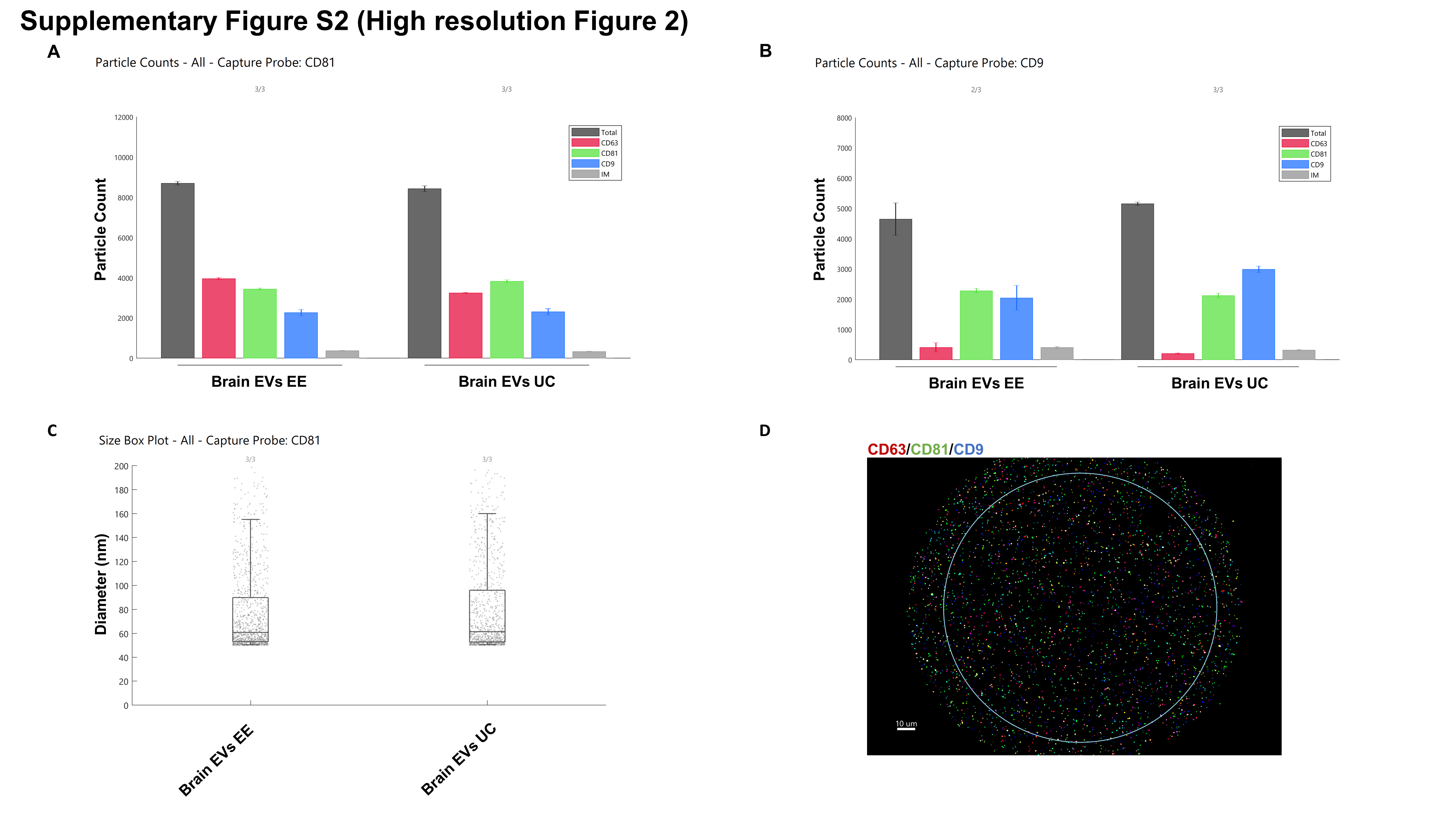

Supplement: Supplementary file 1 [file cells-12-01623-s001.zip › Figure S2.tif]

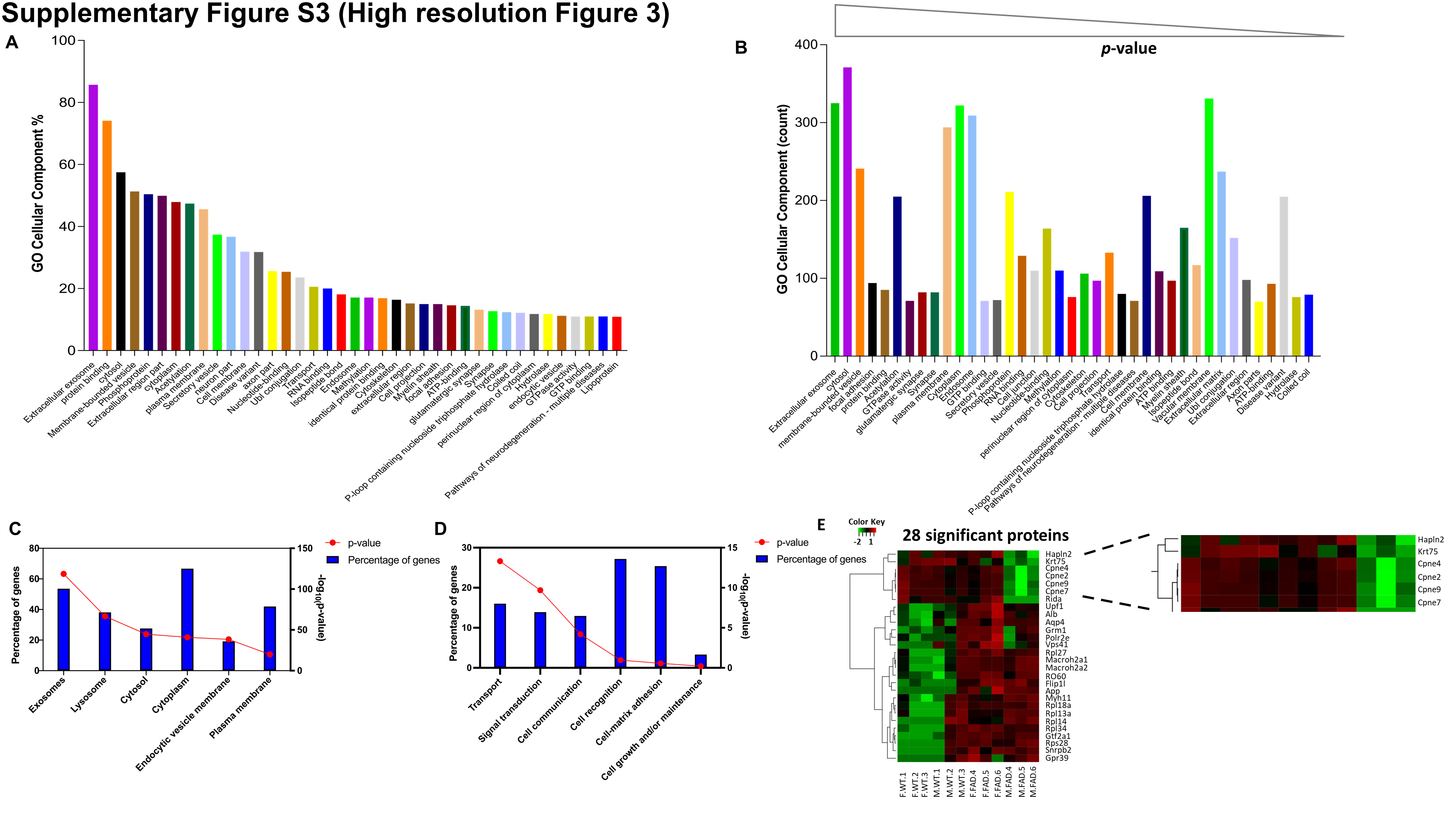

Supplement: Supplementary file 1 [file cells-12-01623-s001.zip › Figure S3.tif]

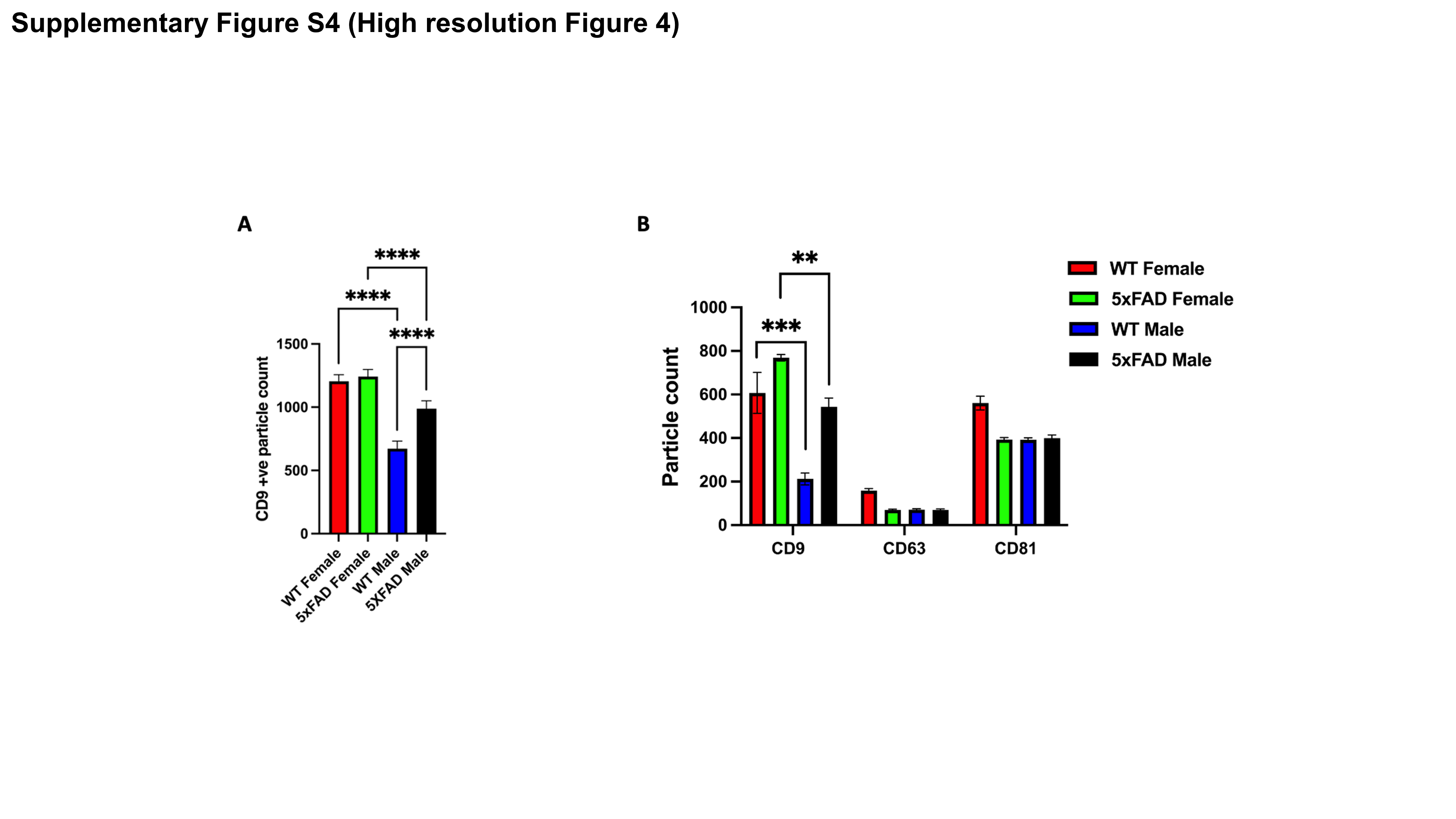

Supplement: Supplementary file 1 [file cells-12-01623-s001.zip › Figure S4.tif]

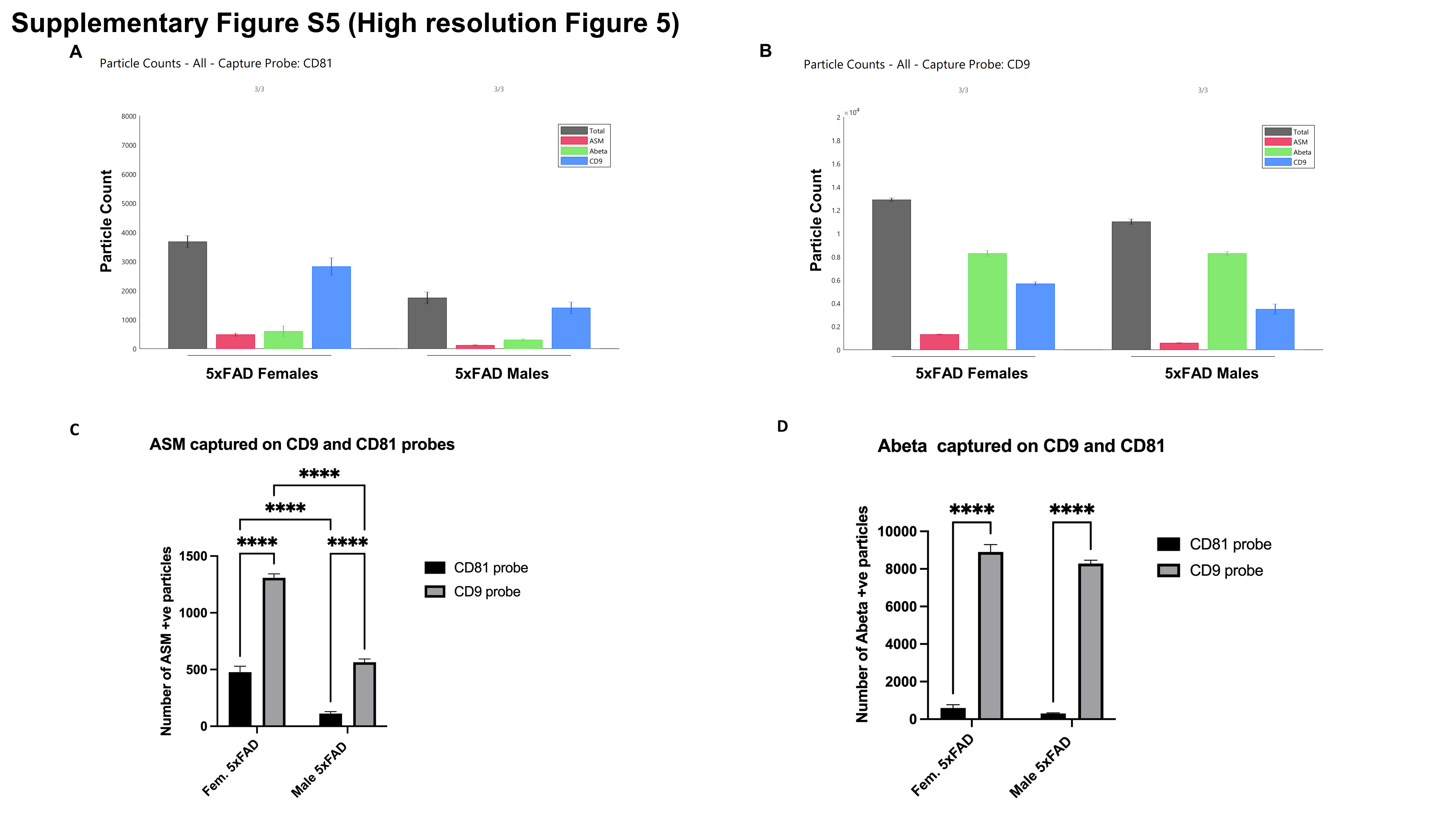

Supplement: Supplementary file 1 [file cells-12-01623-s001.zip › Figure S5.tif]

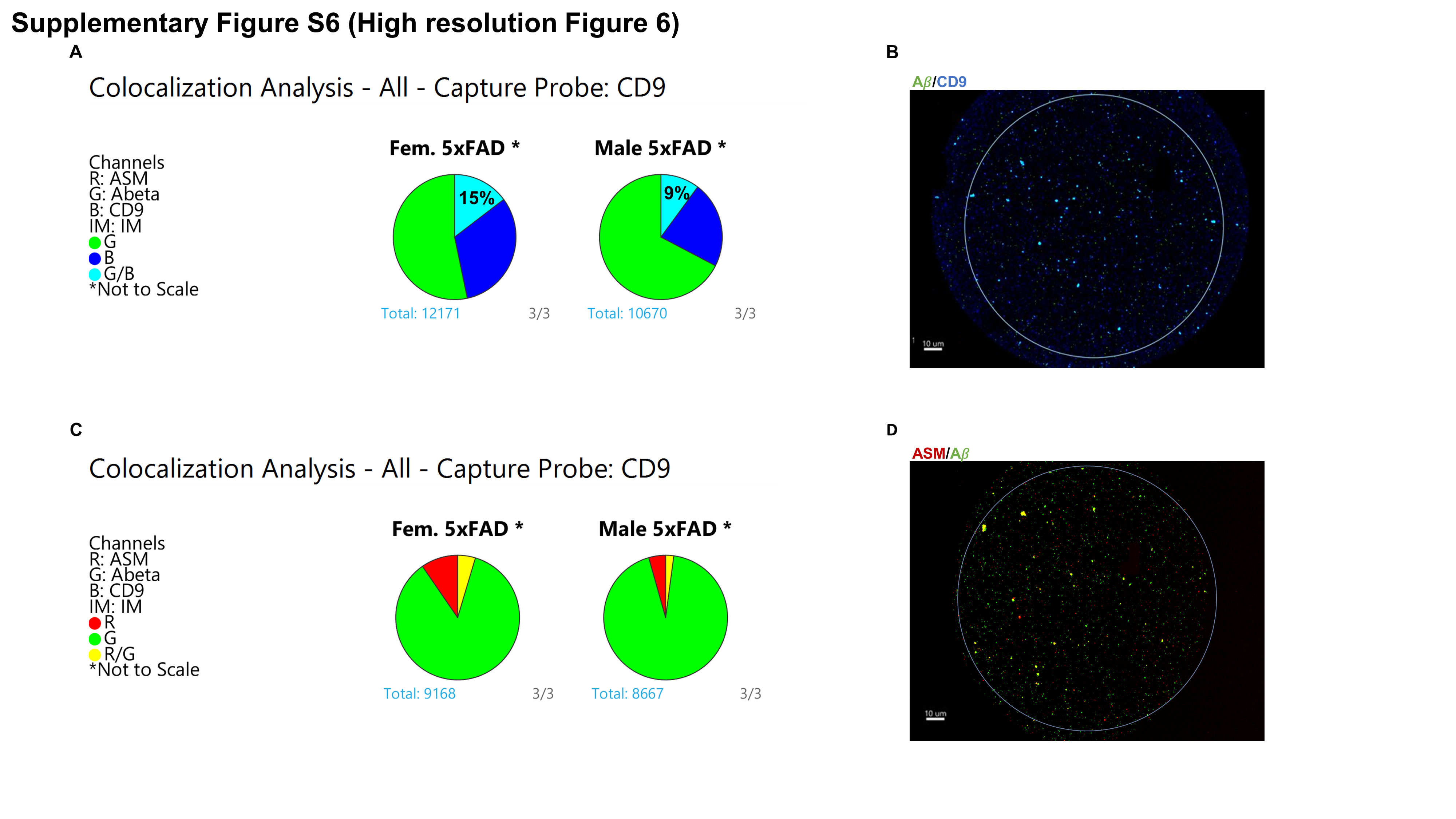

Supplement: Supplementary file 1 [file cells-12-01623-s001.zip › Figure S6.tif]

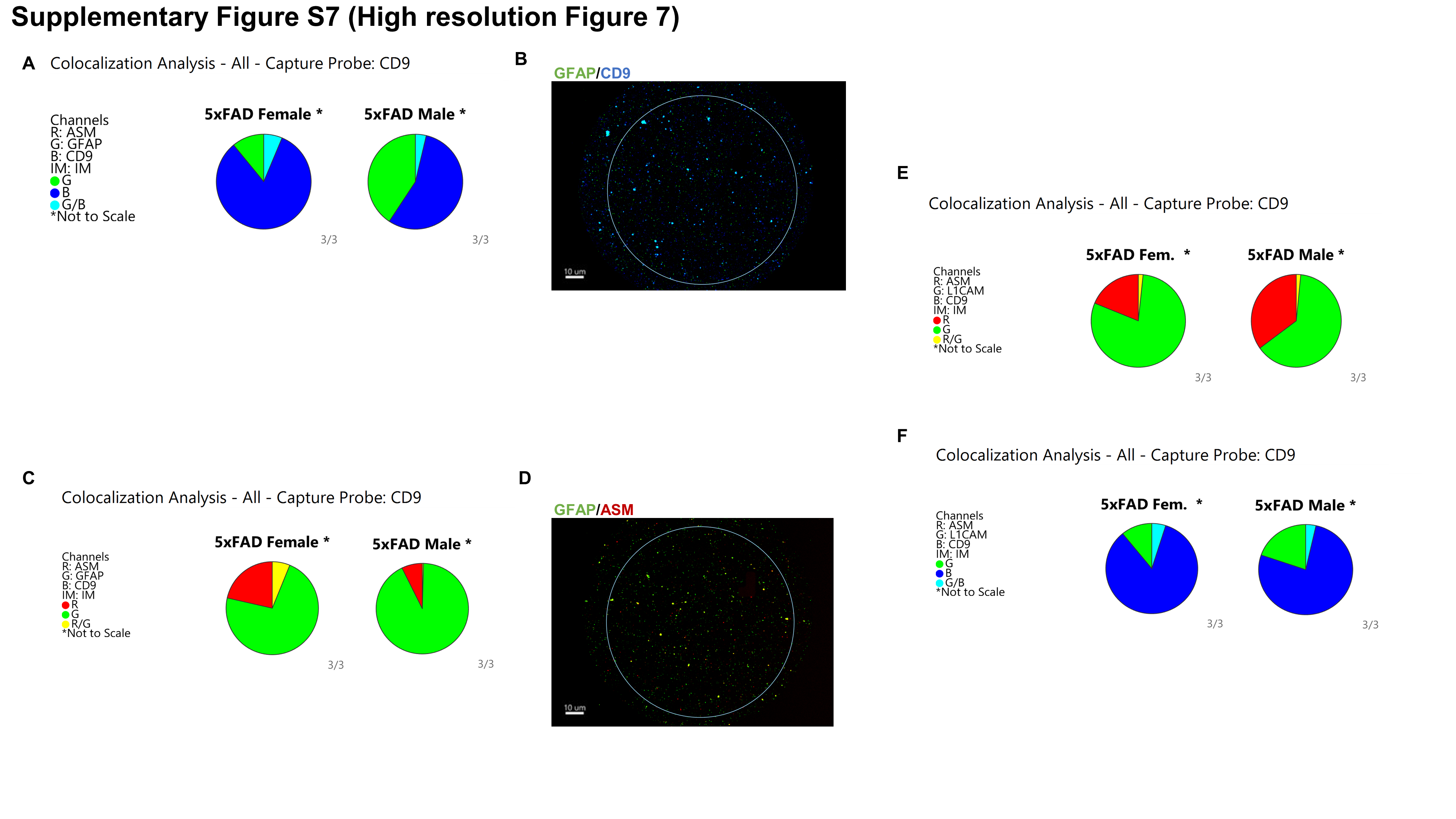

Supplement: Supplementary file 1 [file cells-12-01623-s001.zip › Figure S7.tif]

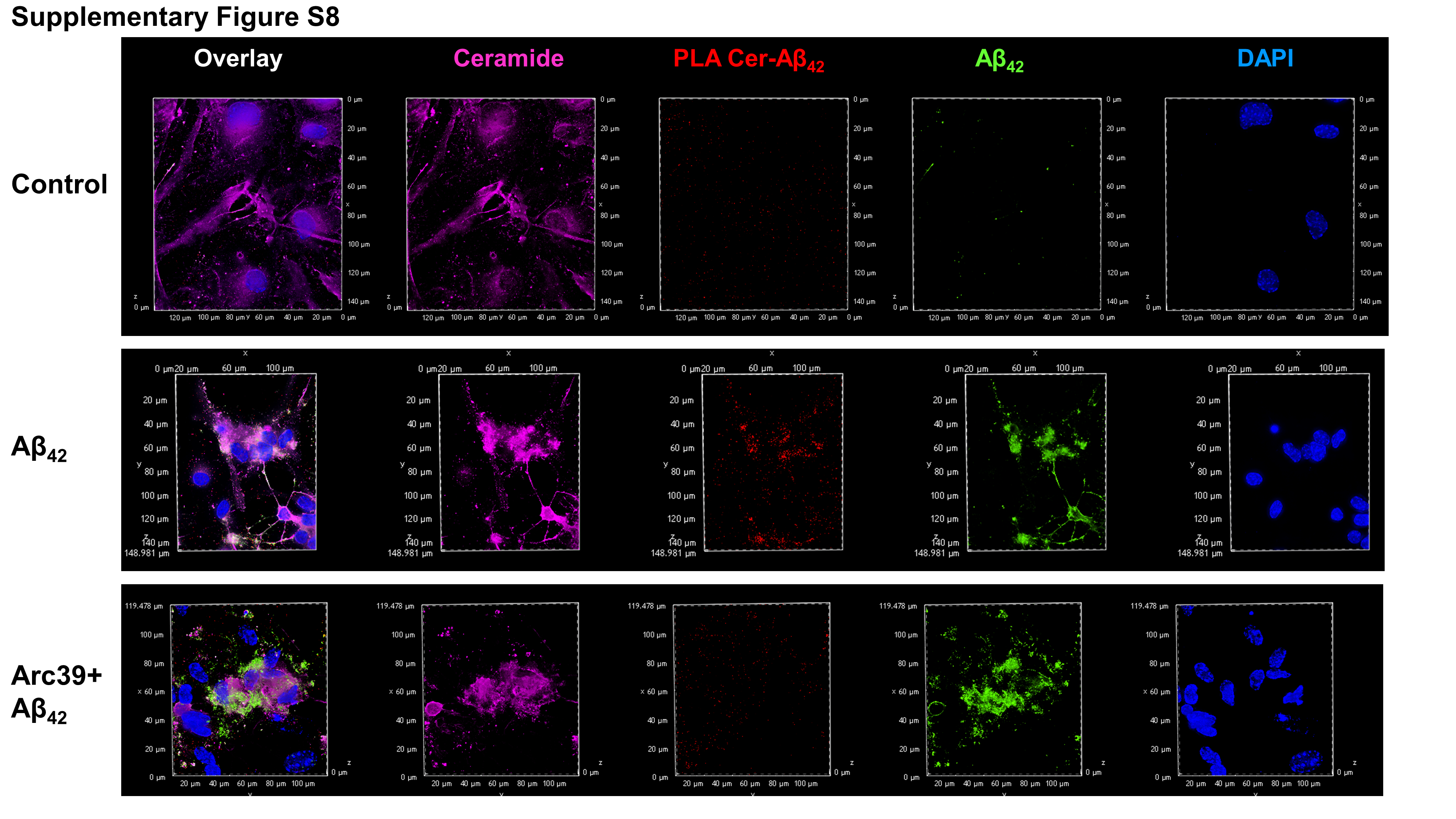

Supplement: Supplementary file 1 [file cells-12-01623-s001.zip › Figure S8.tif]

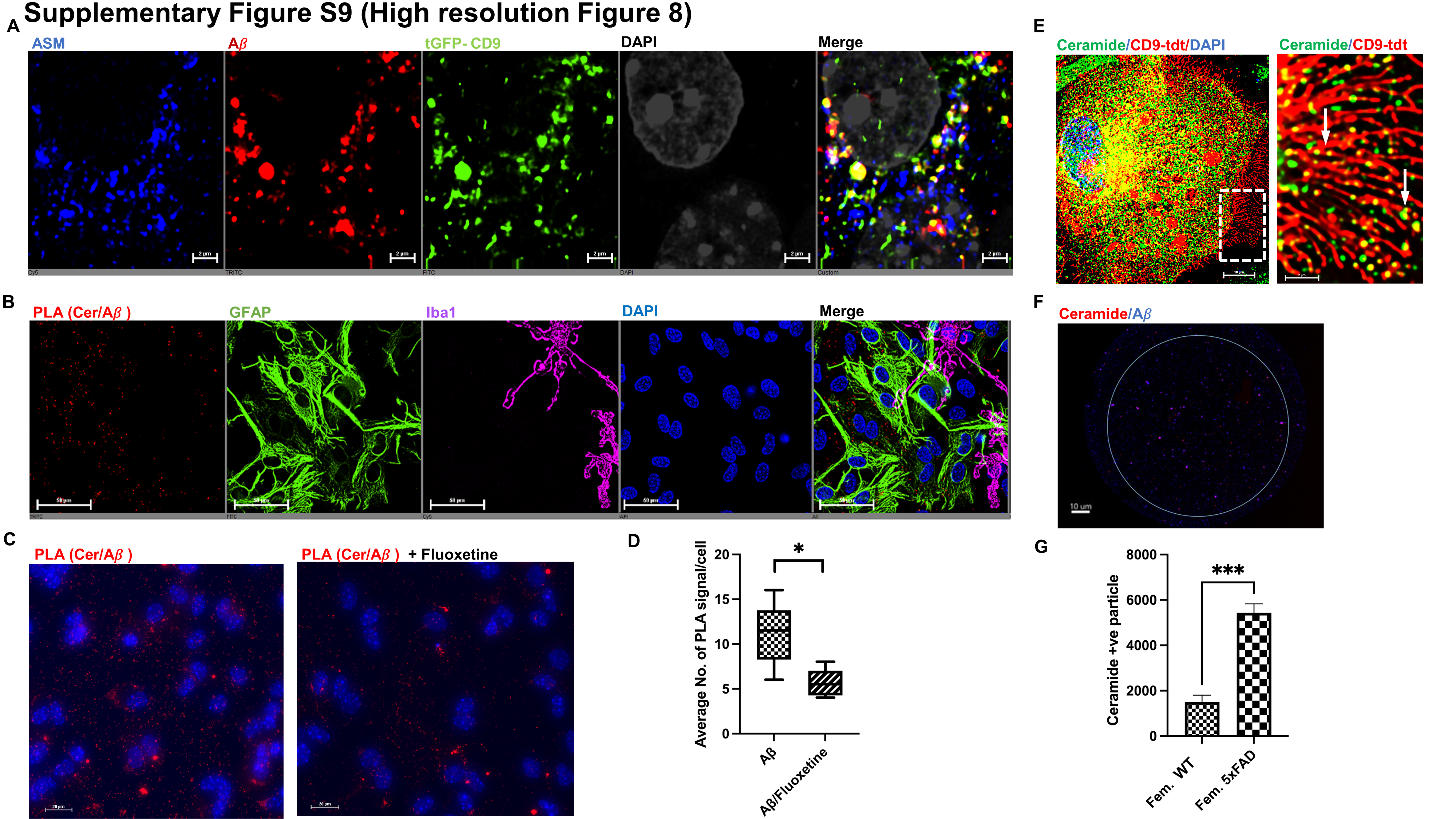

Supplement: Supplementary file 1 [file cells-12-01623-s001.zip › Figure S9.tif]
